# Transcriptome-wide association study of circulating IgE levels identifies novel targets for asthma and allergic diseases

**DOI:** 10.3389/fimmu.2023.1080071

**Published:** 2023-01-30

**Authors:** Kathryn A. Recto, Tianxiao Huan, Dong Heon Lee, Gha Young Lee, Jessica Gereige, Chen Yao, Shih-Jen Hwang, Roby Joehanes, Rachel S. Kelly, Jessica Lasky-Su, George O’Connor, Daniel Levy

**Affiliations:** ^1^ The Population Sciences Branch, National Heart, Lung, and Blood Institute, National Institutes of Health, Bethesda, MD, United States; ^2^ The Framingham Heart Study, Framingham, MA, United States; ^3^ Pulmonary Center, Boston University School of Medicine, Boston, MA, United States; ^4^ Brigham and Women’s Hospital, Channing Division of Network Medicine, Boston, MA, United States

**Keywords:** asthma, allergic diseases, IgE, immunoglobulin, gene expression, immunotherapy, *GCNT1*

## Abstract

Measurement of circulating immunoglobulin E (IgE) concentration is helpful for diagnosing and treating asthma and allergic diseases. Identifying gene expression signatures associated with IgE might elucidate novel pathways for IgE regulation. To this end, we performed a discovery transcriptome-wide association study to identify differentially expressed genes associated with circulating IgE levels in whole-blood derived RNA from 5,345 participants in the Framingham Heart Study across 17,873 mRNA gene-level transcripts. We identified 216 significant transcripts at a false discovery rate <0.05. We conducted replication using the meta-analysis of two independent external studies: the Childhood Asthma Management Program (n=610) and the Genetic Epidemiology of Asthma in Costa Rica Study (n=326); we then reversed the discovery and replication cohorts, which revealed 59 significant genes that replicated in both directions. Gene ontology analysis revealed that many of these genes were implicated in immune function pathways, including defense response, inflammatory response, and cytokine production. Mendelian randomization (MR) analysis revealed four genes (*CLC*, *CCDC21*, *S100A13*, and *GCNT1*) as putatively causal (*p*<0.05) regulators of IgE levels. *GCNT1* (beta=1.5, *p*=0.01)—which is a top result in the MR analysis of expression in relation to asthma and allergic diseases—plays a role in regulating T helper type 1 cell homing, lymphocyte trafficking, and B cell differentiation. Our findings build upon prior knowledge of IgE regulation and provide a deeper understanding of underlying molecular mechanisms. The IgE-associated genes that we identified—particularly those implicated in MR analysis—can be explored as promising therapeutic targets for asthma and IgE-related diseases.

## Introduction

Immunoglobulin E (IgE) is an antibody produced by B cells located in lymph nodes in response to antigenic stimuli and its production requires T helper type 2 (Th2) cells (1). Once released into the circulation, IgE contributes to immunity to respiratory viruses and parasites and protects against venom toxin exposure (2, 3). IgE also plays a role in disease processes related to allergic asthma, allergic rhinitis, atopic dermatitis, and food allergies (4). According to recent estimates from the World Health Organization, asthma affected 300 million people worldwide in 2012 and this number is projected to increase to 400 million by 2025 (4). Given the widespread burden of IgE-mediated allergic diseases, investigating the maladaptive role of IgE in immune responses may highlight promising therapies for asthma and related conditions.

Genome-wide association studies (GWAS) have identified single nucleotide polymorphisms (SNPs) at the *STAT6, FCER1A, IL13, IL4/RAD50*, and the major histocompatibility complex (MHC) loci that are associated with circulating IgE concentrations (5–8). Investigating the transcriptomic signature of IgE concentration may shed light on molecular regulatory mechanisms (9–11). Virkud et al. examined gene expression networks in whole-blood in two independent asthma populations and replicated 31 transcripts associated with serum total IgE (12). To date, however, there have been no published large-scale transcriptome-wide association studies (TWAS) of circulating IgE concentration. While most of the current literature has focused on certain aspects of IgE-related gene regulatory networks, our study was designed to provide a more comprehensive framework for understanding the molecular regulation of IgE by integrating TWAS of IgE with GWAS of IgE and IgE-related diseases.

In this study, we hypothesized *a priori* that IgE-associated transcriptomic changes impact IgE regulation, which in turn play a role in the pathology of IgE-related diseases, such as asthma and allergic diseases. First, we performed a discovery TWAS of IgE in 5345 Framingham Heart Study (FHS) participants. To validate our results, we conducted replication based on the meta-analysis of two independent external studies: the Childhood Asthma Management Program (CAMP) and the Genetic Epidemiology of Asthma in Costa Rica Study (GACRS). We then reversed the discovery and replication sets. Second, we conducted Mendelian randomization (MR) to determine the direction of effect and infer causal relations between gene expression and circulating IgE levels. Two-sample MR analyses were then used to infer causal relations between IgE-related gene expression and IgE-related diseases, including asthma and allergy, by linking genetic variants associated with gene expression (i.e. *cis-*eQTLs) with GWAS of asthma and allergy, respectively (13). By exploring the multidimensional interrelations of gene expression and circulating IgE levels, we provide a deeper understanding of the molecular pathways underlying IgE regulation and highlight promising therapeutic targets for IgE-related diseases.

## Materials and methods

### Discovery in the FHS

#### Study population

**Figure 1:**
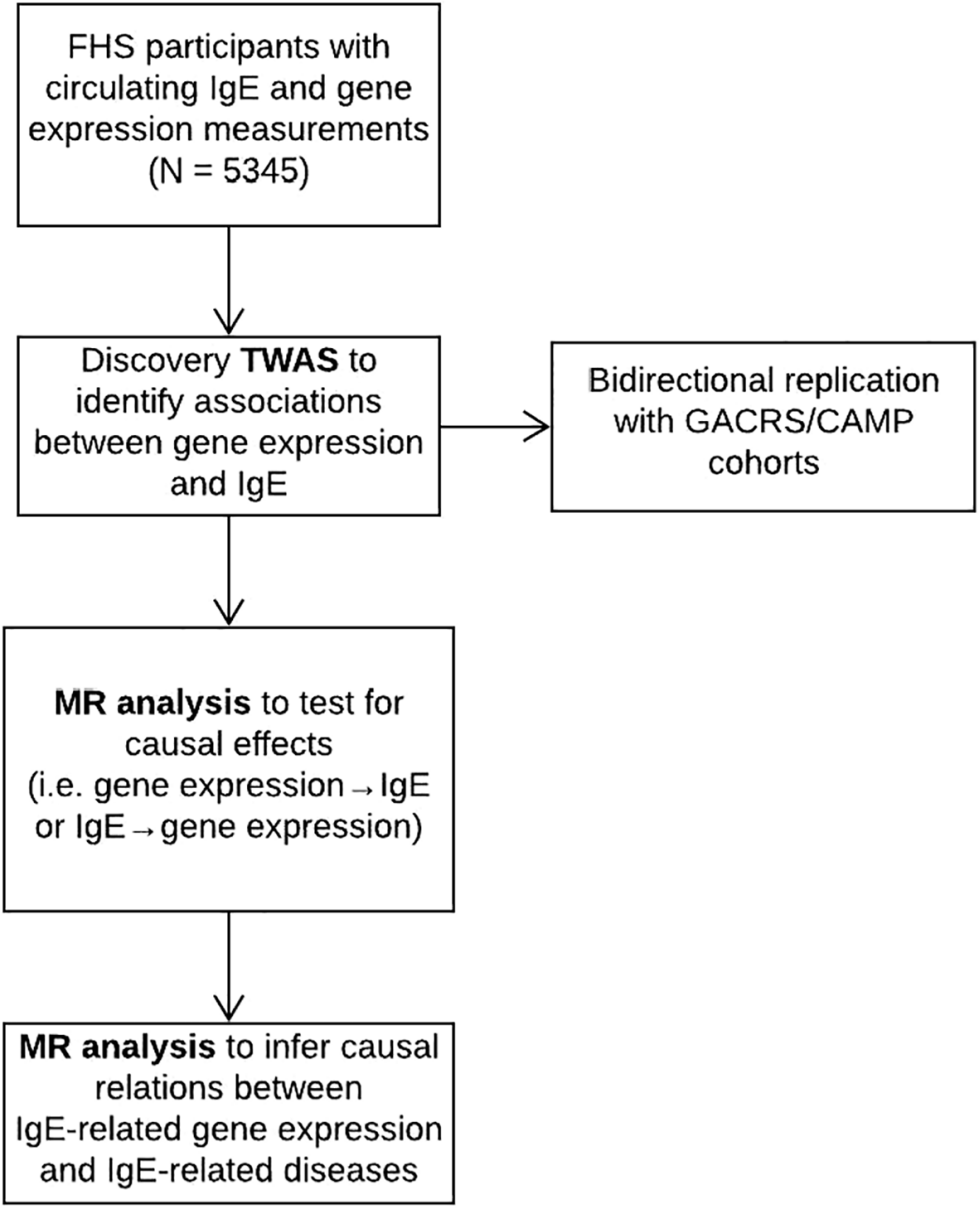
Flowchart of study design.

A flowchart of the study design is displayed in **Figure 1**. The FHS is a community-based study (14). The study sample consisted of 5345 individuals from the FHS Offspring (n=2251) and Third Generation (n=3094) cohorts, in whom IgE levels and gene expression were measured. All the participants from FHS are of European ancestry. The study protocol was approved by the Institutional Review Board at Boston University Medical Center (Boston, MA). All participants gave informed consent for genetic research.

#### Assessment of IgE levels

Serum total IgE concentration was measured on FHS Offspring (Exam 7: 1998-2001) and Third Generation (Exam 1: 2002-2005) cohort participants. Total IgE measurements were performed using the Phadia Immunocap 100 system, in which an anti-IgE antibody is bound to a solid-phase carrier followed by fluoroenzyme-based quantitative measurement of total IgE with high precision and reproducibility (15).

#### mRNA expression data

Gene expression was measured on FHS Offspring (Exam 8: 2005-2008) and Third Generation (Exam 2: 2008-2011) cohort participants. Whole blood samples (2.5 ml) were collected in PAXgene™ tubes (PreAnalytiX, Hombrechtikon, Switzerland). mRNA expression was profiled using the Affymetrix Human Exon 1.0 ST GeneChip (Santa Clara, CA) platform that includes 18,000 gene-level transcripts. The data normalization was described previously (16).

#### Association of gene expression with IgE levels

A linear mixed model implemented in the *lmekin()* package in R was used to analyze associations between gene expression (RMA value) and serum total IgE concentration after adjusting for age, sex, smoking status (current, former, and never smokers), pack-years, technical covariates including batch effects (16), predicted blood cell fraction (including white blood cells, red blood cells, platelets, lymphocytes, monocytes, and basophils), and family structure. We compared the association of gene expression with IgE (T-statistics) with and without cell count adjustment (**Supplementary Figure 1**). This comparison showed that only eosinophils affect the significant associations of gene expression with IgE. Other cell types had little effect on the results. We performed a secondary analysis further adjusting for eosinophils.

#### GWAS of IgE

There have been no large-scale GWAS of serum IgE concentration published in the past five years. Given the limited availability of up-to-date IgE GWAS, we updated a previous FHS GWAS of IgE concentration (8) using 1000 Genomes imputation. We characterized statistical associations between genome-wide polymorphisms and variation of serum IgE concentration using a linear mixed regression model. The updated GWAS included 7252 FHS participants from three cohorts: the FHS Original cohort (Exam 24; 1995-1998; n=495), Offspring cohort (Exam 7; 1998-2001; n=3003), and Third Generation cohort (Exam 1; 2002-2005; n=3764). DNA samples of the FHS participants who gave consent for genomic studies were genotyped using the Affymetrix 550K array (Santa Clara, CA). We applied quality control criteria of 95% call rate, 1×10^-6^ p-value of Hardy-Weinberg equilibrium, and minor-allele-frequency. After applying the quality-control approved genotyping, we generated imputed whole-genome polymorphism panels using the MACH platform and applied the 1000 Genomes phase 1 platform as the reference library. For the current association analysis, we tested for statistical association assuming additive influence of polymorphisms and required an imputation quality of 20% or higher.

#### Mendelian randomization analysis

We used a two-stage least squares (2SLS) Mendelian randomization (MR) method to estimate the causal relationships between gene expression and IgE measured in 5345 FHS participants. Bi-directional MR analyses were performed to test if expression drives IgE concentration (i.e., mRNA → IgE), using the top *cis-*eQTL for each mRNA as an instrumental variable (IV) (16), or if IgE concentration drives mRNA expression, using the genetic risk score combined by the top six loci from previous IgE GWAS results at P<5×10^-8^ (i.e., IgE → mRNA) (5, 8). The six IgE-associated SNPs that were used in the polygenic risk score include rs2251746 (*FCER1A*), rs1059513 (*STAT6*), rs1295686 (*IL13*), rs2523809 (*HLA-G*), rs2517754 (*HLA-A*), and rs2858331 (*HLA-DQA2*) (5, 8). To determine the strength of the genetic instrument, an F-statistic in a linear regression model was derived from the proportion of variation in the exposure that was explained by the corresponding IV. *cis*-eQTLs with an F-statistic less than 10, indicating a weak instrument, were excluded. We considered an mRNA putatively causal for IgE (i.e., mRNA → IgE) when the MR test for mRNA → IgE was significant (*P_mRNA→IgE_* < 0.05), and IgE → mRNA was not significant (*P_IgE→mRNA_ ≥* 0.05).

Two-sample MR was used to identify putatively causal mRNAs for both asthma and allergic diseases using the MRbase package in R. Estimated associations and effect sizes between SNPs and asthma and allergic diseases were based on UK Biobank GWAS of asthma and allergic diseases (hay fever, allergic rhinitis, or eczema) phenotypes, respectively (13). Using *cis-*eQTLs associated with gene transcripts associated with circulating IgE levels as instrumental variables, MR analyses were used to test if gene expression drives asthma/allergy (i.e., mRNA → asthma/allergy).

#### Pathway analysis

Pathway analysis using Gene Ontology (GO) terms was conducted using the online Gene Set Enrichment Analysis tool (gsea-msigdb.org/gsea/msigdb/annotate.jsp), which determines whether an *a priori* defined gene set shows statistically significant, concordant differences between two biological states. Using an FDR q-value <0.05, we identified key biological pathways among the replicated genes associated with serum IgE concentration.

#### Druggable gene targets

We explored approved or experimental drugs targeting the replicated genes using the *rDGIdb* R package, an R wrapper for The Drug Gene Interaction Database (17).

### Replication

#### Study populations

Details of the replication studies (the Childhood Asthma Management Program (CAMP) (18–20) and the Genetic Epidemiology of Asthma in Costa Rica Study (GACRS)) (21) have been described previously, including the assessment of IgE levels and gene expression profiling (20, 22). CAMP samples are from post-trial long-term follow-up blood draws. Written parental consent and child’s assent were obtained, and the study protocol was approved by the Institutional Review Boards at Hospital Nacional de Niños (San Jose, Costa Rica) and Brigham and Women’s Hospital (Boston, MA).

#### Gene expression profiling

In both CAMP and GACRS whole-blood gene expression profiles were generated with probes from the Illumina HumanHT-12 v4 Expression BeadChip (Illumina, Inc., San Diego, USA) that passed stringent and commonly used quality control (QC) metrics (20). we applied a standard non-specific variance filter to the expression data using the “nsFilter” function from the R package “genefilter” (version 1.52). Probes not annotated with a valid Entrez gene identifier or Human Genome Organization (HUGO) gene symbol and probes with interquartile ranges (IQR) of expression variance below the 50^th^ percentile were removed to select only the most informative probes (22). A single gene was then assigned to each probe by collapsing the all probes for that gene based on the largest IQR of expression variance (20). Expression data were log_2_-transformed and quantile-normalized as a single batch using the “lumiT” and “lumiN” functions, respectively, from the R package “lumi” (version 2.22). Principal components (PCs) of gene expression were generated using the “getPCAFunc” function from the R package “iCheck” (version 0.6).

#### Statistical analysis

In both CAMP and GACRS, independent generalized linear regression models were run to test the association between each gene probe and log_10_transformed IgE concentration as a continuous variable, using the “glmwrapper” function from the iCheck package with adjustment for age, sex, and the first two principal components. The Benjamini-Hochberg method was specified to control the false discovery rate with the q-value set to 0.05. The final dataset in GACRS included 25060 gene probes that passed QC from 326 subjects with available data and suitable samples; in CAMP 24972 gene probes from 610 participants were available. All probes measured in CAMP were also measured in GACRS.

#### Meta-analysis

The results from CAMP and GACRS were meta-analyzed using the inverse normal method to combine p-values from the R package metaRNASeq (23). Analyses were weighted according to the study size.

## Results

### FHS discovery TWAS of IgE levels

**Figure 2:**
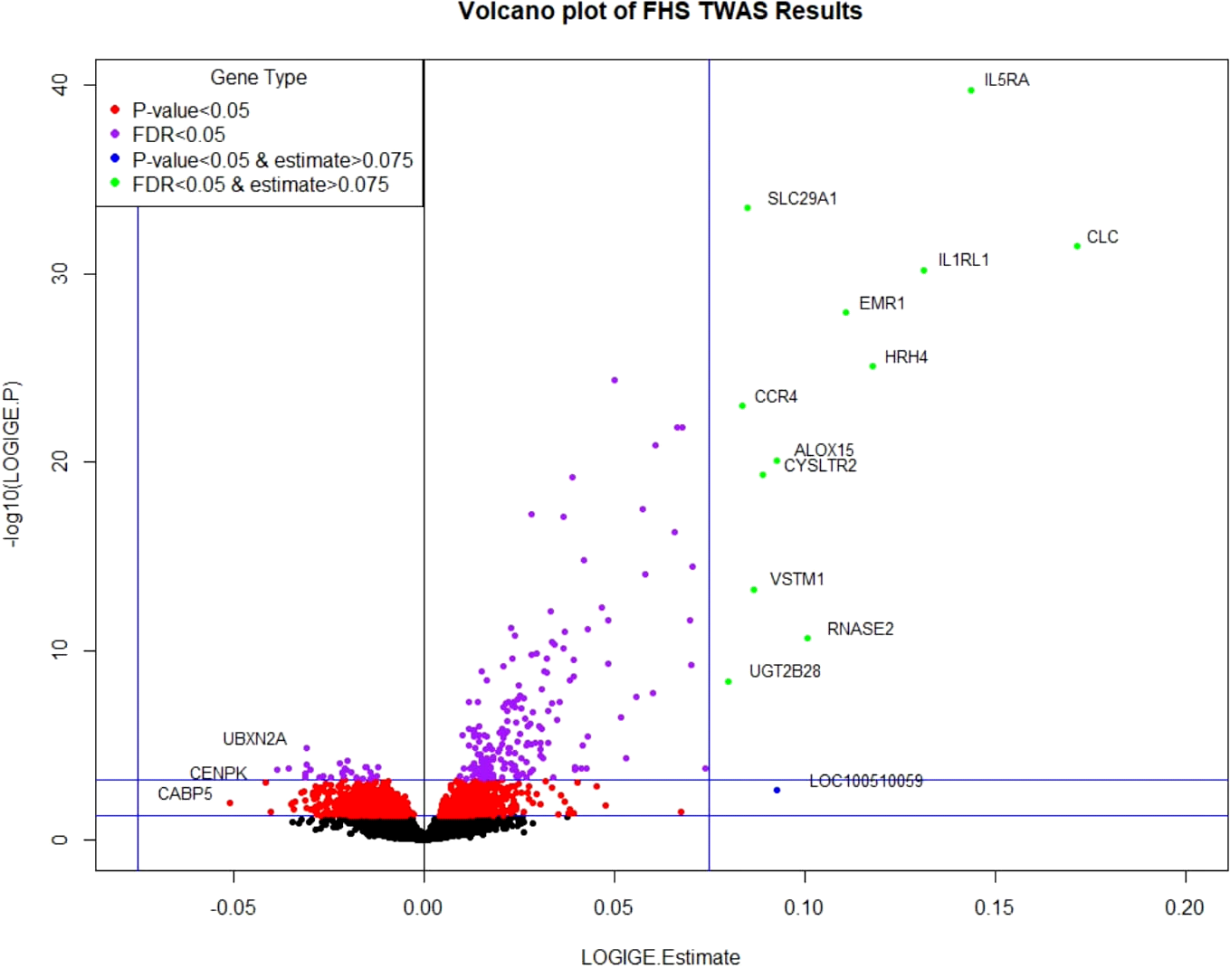
Volcano plot of FHS TWAS results. P-value <0.05 significance threshold is the lower line and P-value <6.09×10^-4^ (which corresponds to an FDR<0.05) is the upper line.

Clinical characteristics of FHS participants (mean age=55 years; 54% women) and the replication cohorts (mean age=20 and 9 years; 37% and 43% women in CAMP and GACRS, respectively) are presented in **Table 1** and **Supplementary Table 1**. In FHS participants, among 17,873 mRNA gene-level transcripts that were available for analysis, 216 were associated with total IgE concentration at a false discovery rate (FDR)<0.05 (**Supplementary Table 2**) and 91 were significant at Bonferroni-corrected p-value threshold of *p*<2.80×10^-6^ (0.05/17,873). The top thirty genes associated with serum IgE concentration are presented in **Table 2**. A volcano plot shows that the vast majority of genes at FDR<0.05 (87.5% or 189/216) had expression levels that were positively associated with IgE (**Figure 2**).

**Table 1:** Participant characteristics of the FHS, GACRS, and CAMP cohorts.

Population	FHS	GACRS	CAMP
**Number (n)**	5345	326	610
**Age (y)**	54.9 (13.3)	9.1 (1.8)	20.4 (2.2)
**Female, n (%)**	2886 (54%)	140 (43%)	226 (37%)
**Log_10_IgE (kU/L)**	1.52 (0.58)	2.5 (0.7)	2.5 (0.6)
**Asthma, n (%)**	406 (7.6%)	326 (100%)	610 (100%)

**Table 2:** Top thirty genes associated with total IgE levels in the FHS.

Gene Symbol	Chr	Beta	SE	P-Value	FDR Value
***IL5RA***	3	0.144	0.011	1.88E-40	3.37E-36
***SLC29A1***	6	0.085	0.007	3.23E-34	2.88E-30
***CLC***	19	0.172	0.014	3.60E-32	2.15E-28
***IL1RL1***	2	0.131	0.011	6.41E-31	2.87E-27
***EMR1***	19	0.111	0.010	1.13E-28	4.04E-25
***HRH4***	18	0.118	0.011	8.13E-26	2.42E-22
***DACH1***	13	0.050	0.005	4.26E-25	1.09E-21
***CCR4***	3	0.084	0.008	1.04E-23	2.32E-20
***TEC***	4	0.067	0.007	1.31E-22	2.46E-19
***SYNE1***	6	0.068	0.007	1.38E-22	2.46E-19
***ADORA3***	1	0.061	0.006	1.32E-21	2.15E-18
***ALOX15***	17	0.093	0.010	7.64E-21	1.14E-17
***CYSLTR2***	13	0.089	0.010	4.43E-20	6.10E-17
***SMPD3***	16	0.039	0.004	5.92E-20	7.55E-17
***IKZF2***	2	0.058	0.007	3.18E-18	3.79E-15
***PRSS33***	16	0.028	0.003	5.30E-18	5.92E-15
***PDE4D***	5	0.037	0.004	7.99E-18	8.40E-15
***CAT***	11	0.066	0.008	4.86E-17	4.82E-14
***SIGLEC8***	19	0.042	0.005	1.52E-15	1.43E-12
***IDO1***	8	0.070	0.009	3.20E-15	2.86E-12
***C2orf46***	2	0.058	0.007	8.65E-15	7.37E-12
***VSTM1***	19	0.087	0.011	5.22E-14	4.24E-11
***CD200R1***	3	0.047	0.006	4.84E-13	3.76E-10
***ARHGAP10***	4	0.033	0.005	7.98E-13	5.94E-10
***CCR3***	3	0.070	0.010	2.38E-12	1.67E-09
***CEBPE***	14	0.048	0.007	2.43E-12	1.67E-09
***GPR114***	16	0.023	0.003	5.64E-12	3.73E-09
***ANXA1***	9	0.043	0.006	7.19E-12	4.59E-09
***C15orf43***	15	0.037	0.005	8.92E-12	5.50E-09
***CAMK1***	3	0.024	0.004	1.50E-11	8.95E-09

**Figure 3:**
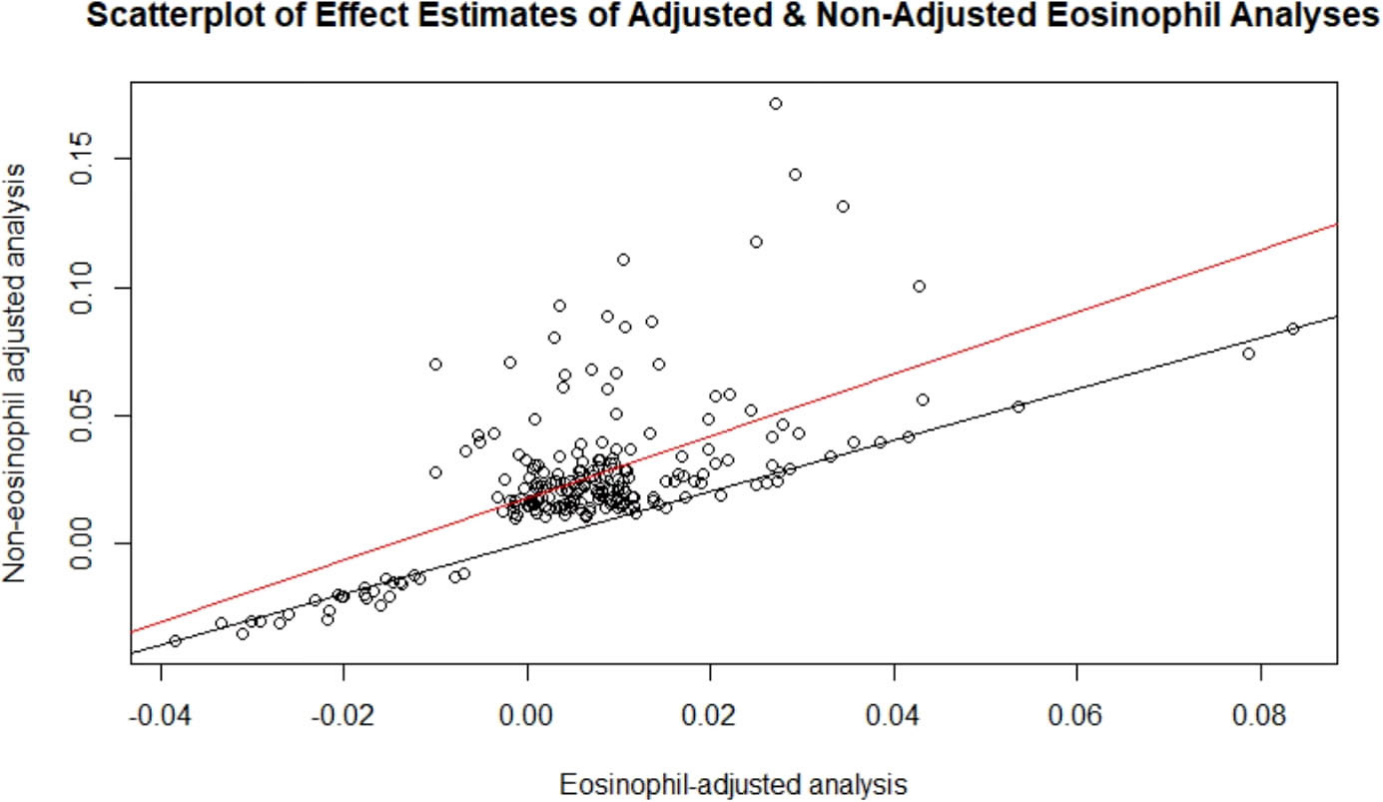
Scatterplot of effect estimates of adjusted and non-adjusted eosinophil analyses. Effect estimates of the 216 FDR-significant genes from the non-eosinophil adjusted analysis plotted against those of the corresponding genes in the eosinophil adjusted analysis. The line of best fit is in red and the y=x line is in black.

After adjusting for eosinophil count (**Supplementary Table 3**), fewer significant genes were identified (12 genes at FDR<0.05, and six at Bonferroni-corrected *p*<2.80×10^-6^). The attenuation of association is because eosinophil count was correlated with IgE level (R=0.24, *p*<1×10^-16^). Eosinophils drive IgE production and reflect the causal pathway of IgE production. Our findings indicate that the mechanisms by which genes influence IgE concentration—and presumably IgE-related diseases—are mediated by eosinophils; thus, adjusting for eosinophils may be an overadjustment. There was concordance of effect estimates (betas) for the IgE-gene expression results with versus without adjustment for eosinophils (R=0.46, *p*<1×10^-16^; **Figure 3**). Thus, we report the results without eosinophil cells adjustment as the primary findings.

### Bi-directional replication

Out of 216 unique transcripts at FDR<0.05 from discovery in FHS, 59 unique transcripts replicated in the meta-analyzed results from GACRS and CAMP (**Table 3**). We defined replication as genes at *p <*2.44×10^-4^ (0.05/205), as only 205 of the 216 significant genes in FHS were available for analysis in the replication cohorts. Forest plots of the top five genes in this replicated gene set are provided in **Supplementary Figure 2**.

**Table 3:** List of replicated gene transcripts (n=59) associated with circulating IgE levels between FHS and meta-analyzed replication cohorts.

Gene Symbol	Gene Name	FHS FDR Value	FHS P-Value	GACRS/CAMP Meta-Analysis P-Value
***IL5RA***	Interleukin 5 Receptor Subunit Alpha	3.37E-36	1.88E-40	8.08E-12
***CLC***	Charcot‐Leyden Crystal Galectin	2.15E-28	3.60E-32	5.00E-24
***EMR1***	Adhesion G Protein‐Coupled Receptor E1 (ADGRE1)	4.04E-25	1.13E-28	1.89E-11
***ADORA3***	Adenosine A3 Receptor	2.15E-18	1.32E-21	2.02E-08
***SMPD3***	Sphingomyelin Phosphodiesterase 3	7.55E-17	5.92E-20	2.14E-16
***PRSS33***	Serine Protease 33	5.92E-15	5.30E-18	2.51E-27
***CAT***	Catalase	4.82E-14	4.86E-17	4.50E-07
***SIGLEC8***	Sialic Acid Binding Ig-Like Lectin 8	1.43E-12	1.52E-15	1.07E-21
***IDO1***	Indoleamine 2,3‐Dioxygenase 1	2.86E-12	3.20E-15	5.50E-19
***VSTM1***	V‐Set & Transmembrane Domain Containing 1	4.24E-11	5.22E-14	1.32E-15
***CD200R1***	CD200 Receptor 1	3.76E-10	4.84E-13	1.23E-05
***ARHGAP10***	Rho GTPase Activating Protein 10	5.94E-10	7.98E-13	9.93E-06
***CCR3***	C‐C motif Chemokine Receptor 3	1.67E-09	2.38E-12	7.13E-15
***CEBPE***	CCAAT Enhancer Binding Protein Epsilon	1.67E-09	2.43E-12	1.99E-28
***GPR114***	Adhesion G Protein-Coupled Receptor G5	3.73E-09	5.64E-12	1.61E-08
***ANXA1***	Annexin A1	4.59E-09	7.19E-12	3.81E-05
***CAMK1***	Calcium/Calmodulin Dependent Protein Kinase I	8.95E-09	1.50E-11	5.10E-14
***RNASE2***	Ribonuclease A Family Member 2	1.16E-08	2.02E-11	4.47E-09
***LGALS12***	Galectin 12	2.54E-08	4.70E-11	8.22E-15
***OLIG2***	Oligodendrocyte Transcription Factor 2	7.58E-08	1.53E-10	4.34E-29
***C6orf97***	Coiled-Coil Domain Containing 170	1.19E-07	2.53E-10	8.94E-05
***CD9***	CD9 Molecule	1.91E-07	4.27E-10	1.19E-06
***P2RY14***	Purinergic Receptor P2Y14	2.26E-07	5.18E-10	2.06E-04
***EEF2K***	Eukaryotic Elongation Factor 2 Kinase	2.66E-07	6.26E-10	2.57E-11
***SRGAP3***	SLIT-ROBO Rho GTPase Activating Protein 3	5.07E-07	1.25E-09	2.24E-09
***INPP1***	Inositol Polyphosphate‐1‐Phosphatase	5.08E-07	1.28E-09	3.49E-14
***CCL23***	C‐C Motif Chemokine Ligand 23	8.26E-07	2.13E-09	9.65E-25
***TRERF1***	Transcriptional Regulating Factor 1	1.23E-06	3.22E-09	5.43E-08
***CD24***	CD24 Molecule	5.68E-06	1.65E-08	1.20E-04
***FBP1***	Fructose-Bisphosphatase 1	7.01E-06	2.08E-08	7.75E-08
***PNPLA6***	Patatin Like Phospholipase Domain Containing 6	1.46E-05	4.81E-08	6.57E-07
***SLC4A8***	Solute Carrier Family 4 Member 8	1.46E-05	4.91E-08	1.53E-04
***GAPT***	GRB2 Binding Adaptor Protein, Transmembrane	1.51E-05	5.16E-08	6.99E-06
***SLC16A14***	Solute Carrier Family 16 Member 14	2.74E-05	1.03E-07	7.63E-08
***ARHGEF6***	Rac/Cdc42 Guanine Nucleotide Exchange Factor 6	3.80E-05	1.45E-07	1.15E-04
***SIGLEC10***	Sialic Acid Binding Ig Like Lectin 10	4.01E-05	1.55E-07	6.95E-08
***DSC2***	Desmocollin 2	7.50E-05	3.02E-07	1.16E-06
***BACE2***	Beta-Secretase 2	2.15E-04	9.61E-07	2.96E-09
***GPR44***	Prostaglandin D2 Receptor 2	2.66E-04	1.21E-06	1.29E-21
***THBS4***	Thrombospondin 4	3.39E-04	1.59E-06	1.93E-11
***OLIG1***	Oligodendrocyte Transcription Factor 1	5.89E-04	3.03E-06	1.88E-20
***VLDLR***	Very Low Density Lipoprotein Receptor	1.20E-03	6.71E-06	5.59E-05
***PLIN2***	Perilipin 2	1.39E-03	8.11E-06	1.42E-05
***ACACB***	Acetyl-CoA Carboxylase Beta	1.57E-03	9.30E-06	1.41E-08
***FAM124B***	Family With Sequence Similarity 124 Member B	2.41E-03	1.56E-05	3.22E-09
***CYP4F12***	Cytochrome P450 Family 4 Subfamily F Member 12	2.47E-03	1.61E-05	3.26E-13
***PAPSS1***	3’-Phosphoadenosine 5’-Phosphosulfate Synthase 1	4.95E-03	3.41E-05	9.11E-06
***SLC24A3***	Solute Carrier Family 24 Member 3	6.67E-03	4.74E-05	1.34E-05
***IL17RB***	Interleukin 17 Receptor B	1.18E-02	9.20E-05	1.60E-06
***GFI1B***	Growth Factor Independent 1B Transcriptional Repressor	1.55E-02	1.23E-04	6.42E-07
***TRIB1***	Tribbles Pseudokinase 1	1.64E-02	1.35E-04	2.53E-05
***CD63***	CD63 Molecule	1.64E-02	1.36E-04	1.66E-04
***SUSD1***	Sushi Domain Containing 1	1.72E-02	1.45E-04	6.13E-05
***FCRLA***	Fc Receptor Like A	1.91E-02	1.71E-04	8.03E-05
***BRI3BP***	BRI3 Binding Protein	2.04E-02	1.94E-04	6.87E-06
***GPR137B***	G Protein-Coupled Receptor 137B	2.23E-02	2.15E-04	3.10E-05
***CACNG6***	Calcium Voltage-Gated Channel Auxiliary Subunit Gamma 6	2.46E-02	2.43E-04	7.74E-05
***SPNS3***	Sphingolipid Transporter 3 (Putative)	3.76E-02	4.12E-04	2.76E-18
***C3AR1***	Complement C3a Receptor 1	4.18E-02	4.81E-04	1.04E-06

We performed reverse replication with the meta-analysis of GACRS/CAMP as the discovery set and FHS as the replication set. From the meta-analysis of GACRS/CAMP, we identified 135 unique transcripts associated with total IgE levels at FDR<0.05. Among these, 114 transcripts mapping to 112 unique genes were available in FHS (*TRERF1* and *ACOT11* were each linked to two separate transcripts). We defined replication as *p*<4.39×10^-4^ (0.05/114); all 114 significant transcripts from discovery in GACRS/CAMP replicated in FHS (**Supplementary Table 4**). Furthermore, all 59 genes that replicated in GACRS/CAMP based on FHS discovery were within the 114 replicated gene set using GACRS/CAMP as discovery—i.e., 59 genes demonstrated bi-directional replication, demonstrating the robustness of association signals (**Table 3**).

Of note, the sample size in FHS (N=5345) is much larger than in GACRS and CAMP (N=936 in total). The larger sample size provides greater power to identify significant results in FHS than in the other two cohorts at a given FDR threshold (216 vs. 135 significant transcripts at FDR<0.05).

### Gene ontology

Gene ontology analysis was performed on the 59 genes that bi-directionally replicated between FHS and GACRS/CAMP. Multiple genes from this gene set were associated with pathways involved in inflammation and other immune system responses (**Table 4**). We also checked if any of the 59 replicated genes were approved or experimental drugs targets. Among the 59 genes, 17 mapped to 86 drug compounds from multiple drug database sources (**Supplementary Table 5**).

**Table 4:** Gene ontology defined biological processes associated with total IgE levels in the bi-directionally replicated transcripts between the FHS and GACRS/CAMP cohorts (n=59).

GO biological process	# Genes in GO group	P-Value	FDR Value
**Inflammatory Response**	11	1.30E-08	1.24E-04
**Defense Response**	13	1.69E-06	2.69E-03
**Cytokine Production**	9	2.46E-06	3.35E-03
**Regulation of Leukocyte Migration**	5	1.10E-05	6.41E-03
**Myeloid Leukocyte Migration**	5	1.63E-05	8.35E-03
**Granulocyte Migration**	4	6.10E-05	2.33E-02
**Gliogenesis**	5	7.92E-05	2.70E-02
**Leukocyte Proliferation**	5	9.17E-05	3.02E-02
**Regulation of T Cell Tolerance Induction**	2	1.05E-04	3.25E-02
**Leukocyte Migration**	6	1.09E-04	3.26E-02
**IL5 Pathway**	2	1.82E-04	4.28E-02
**T Cell Proliferation**	4	1.84E-04	4.28E-02

### Mendelian randomization for total IgE levels

**Figure 4:**
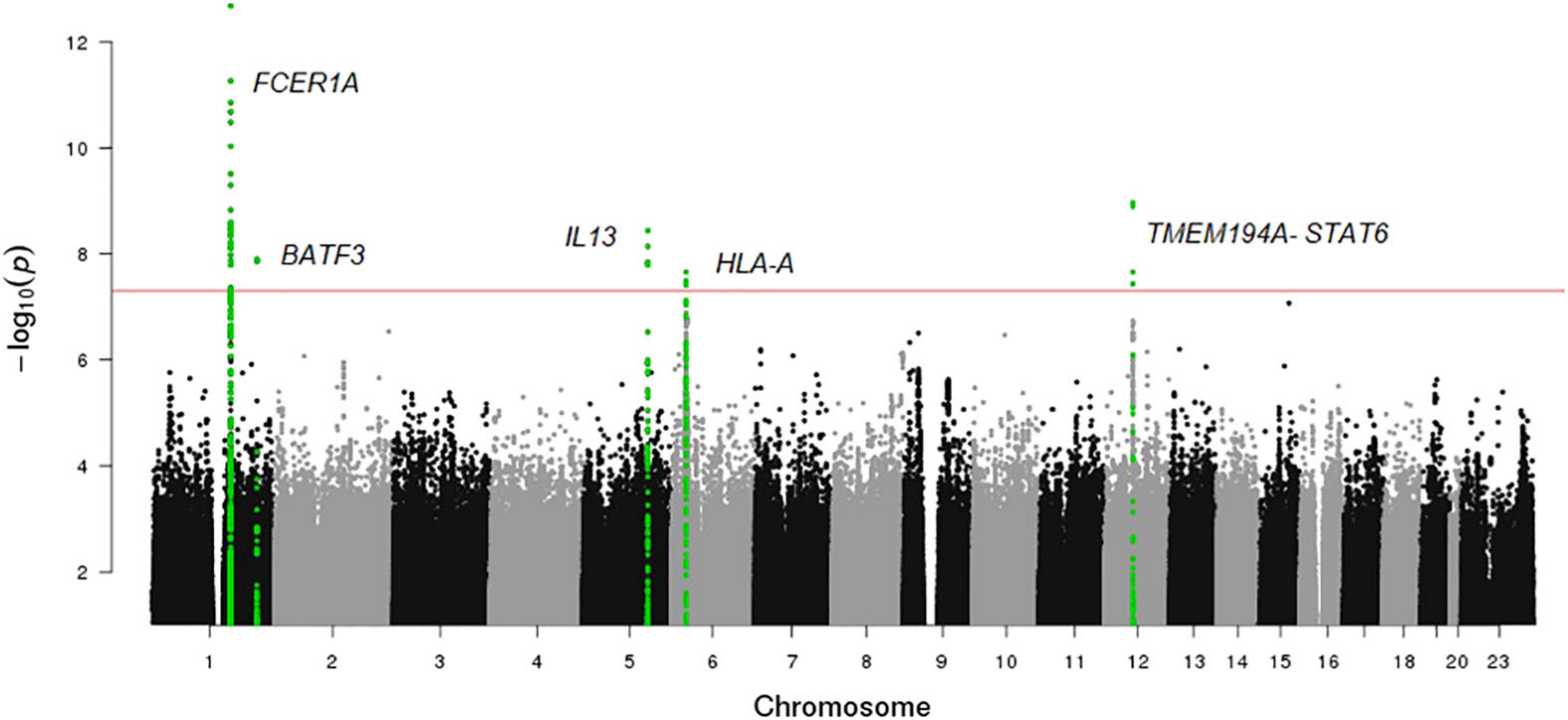
Manhattan plot of FHS GWAS results. The horizontal line shows the threshold for genome wide significance (P-value <5×10^-8^).

**Figure 5:**
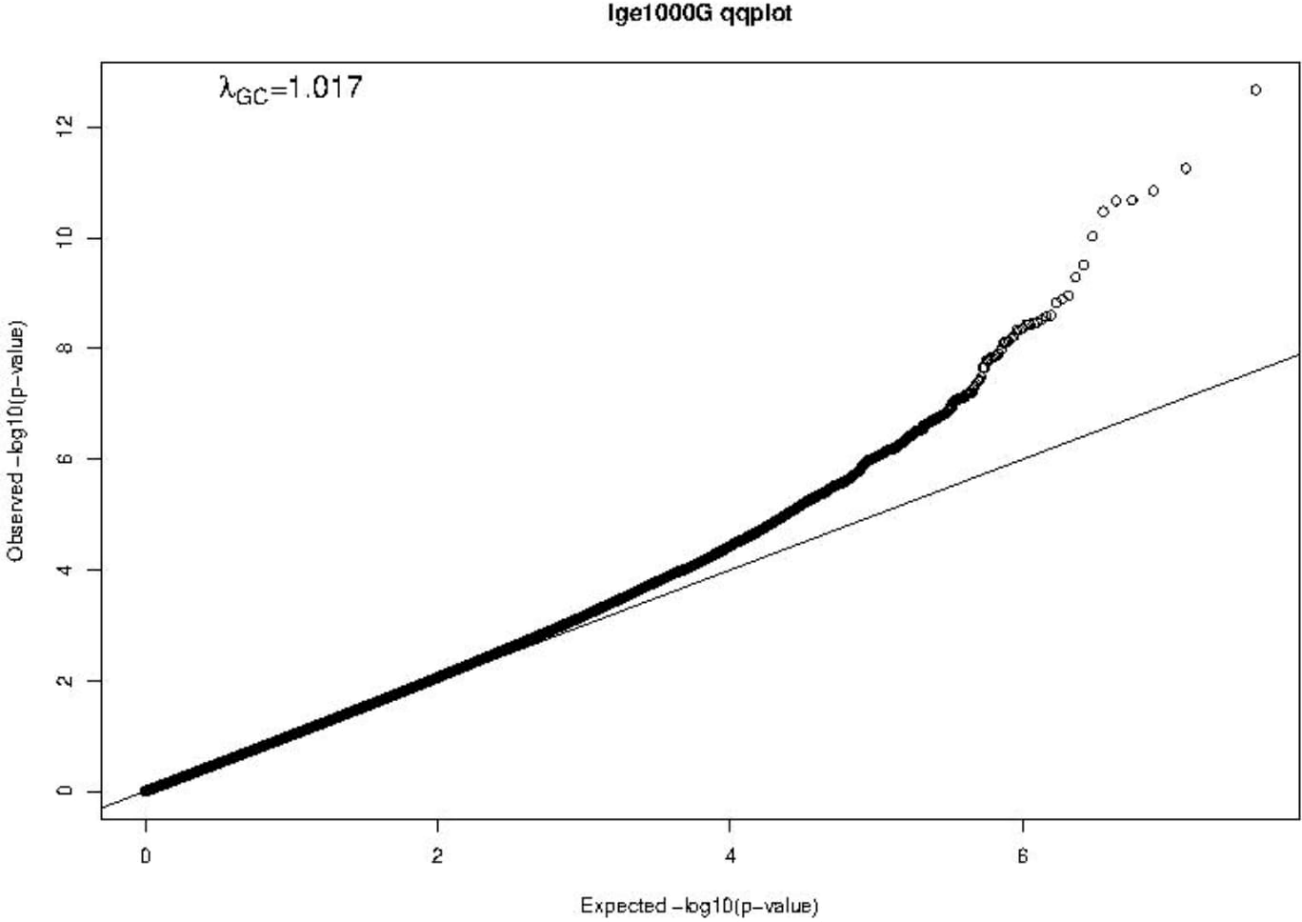
Q-Q plot of FHS GWAS results. The Q-Q plot shows observed vs. expected −log10(P-values) of the GWAS results. The straight line represents the SNP distribution under the null hypothesis.

A Manhattan plot and a Q-Q plot (lambda 1.017) displaying the updated FHS IgE GWAS results are provided in **Figures 4** and **5**, respectively. A list of significant SNPs (*p*<5×10^-8^) from the updated IgE GWAS is reported in **Supplementary Table 6**. Among the 216 FDR-significant genes identified in FHS, 185 genes had suitable *cis-*eQTLs for the MR analysis. We conducted bi-directional MR to test causal relations between expression levels of the 185 genes and circulating IgE levels. We identified four genes—*CLC*, *CCDC21*, *S100A13*, and *GCNT1*—as putatively causal for IgE at *P_mRNA→IgE_* < 0.05 using the top *cis-*eQTL for each gene as an instrument variable (**Table 5**).

**Table 5:** Bi-directional MR results for genes putatively causal for IgE levels at p<0.05 (n=4).

Gene Symbol	SNP	Chr	Beta _(mRNA→IgE)_	SE _(mRNA→IgE)_	P-Value _(mRNA→IgE)_	Beta _(IgE→mRNA)_	SE _(IgE→mRNA)_	P-Value _(IgE→mRNA)_
***CCDC21***	rs869683	1	0.571	0.197	3.67E-03	-0.005	0.024	8.46E-01
***S100A13***	rs9661993	1	-0.166	0.062	7.35E-03	-0.097	0.057	9.15E-02
***GCNT1***	rs11144929	9	1.503	0.606	1.32E-02	-0.018	0.03	5.62E-01
***CLC***	rs17709471	19	0.467	0.198	1.80E-02	-0.061	0.082	4.58E-01

Additionally, we performed reverse MR using the top six SNPs from IgE GWAS combined as a polygenic risk score to test if IgE level affected gene expression levels. None of the four genes from forward MR were significant in reverse MR (*P_IgE→mRNA_* ≥ 0.05) (**Table 5**), suggesting a stronger likelihood that gene expression drives changes in IgE levels rather than IgE levels driving gene expression.

### Mendelian randomization for IgE-related diseases: Asthma and allergic diseases

We conducted two-sample MR testing to infer a causal relation between IgE-related gene expression and IgE-related diseases, specifically asthma and allergic diseases. We identified 70 genes that were putatively causal for asthma and 71 genes that were putatively causal for allergic diseases at a Bonferroni-corrected p-value threshold of *p*<2.70×10^-4^ (0.05/185) (**Table 6**; **Supplementary Table 7**). In comparing the MR results of asthma to those of allergic diseases, the vast majority of putatively causal genes (N=68) overlapped, which is to be expected given that asthma and allergic diseases are IgE-related (**Table 6**).

**Table 6:** MR results for genes putatively causal for asthma at Bonferroni-corrected p<2.70×10^-4^ (n=70).

Gene Symbol	Transcript #	Method	nsnp	Beta	SE	P-Value
***GPI***	3829687	Wald ratio	1	23.67	0.62	<1E-300
***PGD***	2319802	Wald ratio	1	70.20	1.08	<1E-300
***NDFIP2***	3495076	Wald ratio	1	76.43	1.01	<1E-300
***CYSLTR2***	3489138	Wald ratio	1	21.71	0.30	<1E-300
***GPR114***	3662774	Wald ratio	1	-60.23	0.94	<1E-300
***CPT1A***	3379644	Wald ratio	1	34.88	0.66	<1E-300
***ZNF610***	3840164	Wald ratio	1	-51.06	0.72	<1E-300
***ABTB2***	3368940	Wald ratio	1	48.78	0.67	<1E-300
***CD9***	3402315	Wald ratio	1	26.12	0.55	<1E-300
***STK17A***	2999485	Wald ratio	1	42.04	0.71	<1E-300
***SMPD3***	3696317	Wald ratio	1	-59.35	1.01	<1E-300
***GCNT1***	3175494	Wald ratio	1	58.12	0.84	<1E-300
***CCR4***	2616131	Wald ratio	1	20.37	0.32	<1E-300
***PRF1***	3293435	Wald ratio	1	40.38	0.93	<1E-300
***ATP8B2***	2360206	Wald ratio	1	47.85	0.71	<1E-300
***ZMYND11***	3231389	Wald ratio	1	-71.58	1.00	<1E-300
***OLIG1***	3918429	Wald ratio	1	96.66	1.41	<1E-300
***FBP1***	3215570	Wald ratio	1	-62.42	1.16	<1E-300
***ORM2***	3186137	Wald ratio	1	-29.95	0.38	<1E-300
***SAMSN1***	3925473	Wald ratio	1	51.39	0.82	<1E-300
***FCRL2***	2439052	Wald ratio	1	-52.09	0.83	<1E-300
***CCDC86***	3332548	Wald ratio	1	58.53	0.80	<1E-300
***KIT***	2727587	Wald ratio	1	86.75	1.15	<1E-300
***PLD3***	3833443	Wald ratio	1	40.25	0.87	<1E-300
***BHLHE40***	2608725	Wald ratio	1	-24.02	0.47	<1E-300
***THUMPD1***	3683783	Wald ratio	1	31.31	0.42	<1E-300
***CLINT1***	2883609	Wald ratio	1	59.72	0.82	<1E-300
***IL5RA***	2660617	Wald ratio	1	44.95	0.62	<1E-300
***IL2RA***	3275729	Wald ratio	1	28.73	0.40	<1E-300
***SRGAP3***	2662087	Wald ratio	1	79.94	1.07	<1E-300
***NUPL1***	3482219	Wald ratio	1	53.59	1.27	<1E-300
***NUP93***	3662265	Wald ratio	1	53.06	0.66	<1E-300
***IL17RB***	2624565	Wald ratio	1	-33.81	0.45	<1E-300
***CRIP1***	3554851	Wald ratio	1	28.22	0.43	<1E-300
***GPR137B***	2386747	Wald ratio	1	-65.16	0.85	<1E-300
***ID2***	2468622	Wald ratio	1	-38.70	0.49	<1E-300
***CLCNKB***	2322264	Wald ratio	1	68.19	0.93	<1E-300
***PLAC4***	3932917	Wald ratio	1	-15.40	0.21	<1E-300
***SYNE1***	2979871	Wald ratio	1	-45.92	1.08	<1E-300
***FCRLA***	2363852	Wald ratio	1	-66.59	0.70	<1E-300
***COBLL1***	2584787	Wald ratio	1	84.94	1.28	<1E-300
***INPP5A***	3272205	Wald ratio	1	-22.45	0.50	<1E-300
***SEMA7A***	3632907	Wald ratio	1	-49.69	1.14	<1E-300
***ADORA3***	2427981	Inverse variance weighted	2	19.38	0.43	<1E-300
***GATA3***	3234277	Wald ratio	1	42.41	0.58	<1E-300
***PMP22***	3746574	Inverse variance weighted	2	30.48	0.82	<1E-300
***P4HA1***	3294159	Wald ratio	1	10.88	0.30	1.23E-288
***KLHL6***	2708066	Wald ratio	1	-21.75	0.65	5.52E-244
***PPM1L***	2650393	Wald ratio	1	-11.22	0.35	5.00E-224
***GRB10***	3050462	Wald ratio	1	28.66	0.94	5.03E-204
***CDK15***	2522916	Wald ratio	1	10.88	0.39	5.54E-175
***BRI3BP***	3436544	Wald ratio	1	31.48	1.14	5.51E-169
***SEMA5A***	2847967	Wald ratio	1	36.84	1.44	3.84E-145
***HRASLS2***	3376512	Wald ratio	1	10.69	0.43	7.30E-139
***PDE8A***	3606034	Wald ratio	1	23.80	1.16	2.96E-93
***CD63***	3457160	Inverse variance weighted	2	-80.22	5.03	3.30E-57
***VKORC1L1***	3005280	Wald ratio	1	22.91	1.44	8.49E-57
***SLC35D1***	2417095	Inverse variance weighted	3	14.53	1.87	8.13E-15
***FMNL3***	3454006	Inverse variance weighted	2	-37.28	5.70	6.10E-11
***TNIK***	2705266	Inverse variance weighted	3	-64.09	9.84	7.23E-11
***VSTM1***	3870449	Inverse variance weighted	3	-6.97	1.17	2.49E-09
***CCL23***	3753985	Inverse variance weighted	2	-36.91	7.31	4.35E-07
***VLDLR***	3160175	Inverse variance weighted	5	33.87	6.73	4.76E-07
***TNFRSF9***	2395146	Inverse variance weighted	2	-12.54	2.60	1.41E-06
***ABCC1***	3649890	Inverse variance weighted	2	-44.86	10.32	1.39E-05
***KLF6***	3274361	Inverse variance weighted	2	-54.40	13.38	4.79E-05
***CASP3***	2796484	Inverse variance weighted	2	-11.03	2.74	5.69E-05
***TEC***	2768396	Inverse variance weighted	3	-22.47	5.73	8.74E-05
***GZMB***	3558375	Inverse variance weighted	2	-13.92	3.61	1.17E-04
***INPP1***	2520113	Inverse variance weighted	2	-25.81	6.83	1.58E-04

*GCNT1*, a putatively causal gene for IgE concentration as implicated in our MR analysis of gene expression in relation to IgE levels (beta=1.503, *p*=0.01; **Table 5**), is also one of the top results in the MR analysis of expression in relation to asthma and allergic diseases (beta=58.12, *p*<1×10^-400^ and beta=58.88, *p*<1×10^-400^, respectively; **Table 6** and **Supplementary Table 7**).

## Discussion

A thorough understanding of the molecular mechanisms underlying the regulation of IgE is essential for developing new therapies for asthma and other IgE-mediated diseases, such as allergic rhinitis, atopic dermatitis, and food allergies. To the best of our knowledge, this is the first large-scale TWAS study of total IgE levels that uses MR to infer causal relations between gene expression and IgE levels. In this study, we identified a transcriptomic signature of IgE consisting of 216 FDR-significant genes from discovery in FHS. Gene ontology analysis of this gene set shows that many of these IgE-related genes are enriched in key pathways related to regulation of immune system processes, defense response, and inflammatory response.

Bi-directional MR analysis revealed four genes (*CLC*, *CCDC21*, *S100A13*, and *GCNT1*) as nominally significant (*P_mRNA→IgE_* < 0.05) causal regulators of IgE concentration without reverse causal effect (*P_IgE->mRNA_* > 0.05), suggesting that individual gene transcripts that are associated with IgE concentration likely contribute causally to IgE regulation. Admittedly, the MR results should be interpreted with caution in the absence of functional validation. Among the four putatively causal genes is *CLC* (Charcot-Leyden crystal galectin), which is overexpressed in eosinophils that are stimulated following binding of IgE (24). Prior studies have identified increased CLC protein levels in induced sputum as a surrogate biomarker of eosinophilic airway inflammation in asthma (25). Another recent study used a humanized mouse model of asthma to demonstrate that administration of CLC protein with house dust mites (HDM) increased human IgE synthesis compared to when HDM was administered alone. The strong association of the protein encoded by *CLC* with IgE concentration, revealed by our TWAS and MR analysis, highlights *CLC* as a key gene and attractive therapeutic target. This association does not persist after adjusting for eosinophil count, likely because the mechanisms by which *CLC* genetic variants and expression influence IgE concentration—and presumably asthma and allergic diseases—are mediated by eosinophils.

An emerging area of interest in immunology in recent years is the effects on immunity and disease susceptibility of glycosylation of lipid or protein molecules by glycans such as *GCNT1* (glucosaminyl (N-acetyl) transferase 1) (26). *GCNT1* is a glycosyltransferase involved in pathways related to metabolism of proteins, and it has several functions involved in immune response. One recent study demonstrated that the protein product of *GCNT1*, core 2 ß1,6-*N*-acetylglucosaminyltransferase-I (C2GlcNAcT-I), is necessary not only for the synthesis of P-selectin ligands in neutrophils and T helper 1 (Th1) cells but also for the homing of Th1 cells into sites of inflammation (27). Additional roles of *GCNT1* include partially controlling lymphocyte trafficking into lymph nodes and regulating B cell differentiation *via* formation and extension of core 2 O-glycans (28, 29). These functions are critical to understanding the relations of *GCNT1* to IgE concentration given that B cells produce IgE.

Interestingly, a recent knockout study found that *GCNT1* deficient mice have neutrophilia and increased susceptibility to tuberculosis infection. The increased susceptibility of *GCNT1* deficient mice to infection was largely driven by exacerbated neutrophil counts, which led to lung lesions, inflammation, and other pathologic features in the lungs of affected mice (30). This link between *GCNT1* and neutrophilia is relevant to studying the regulation of IgE as other studies have shown elevated serum IgE levels to be associated with neutrophilic asthma (31). Therefore, it is possible that a deficiency, or more broadly an alteration, in *GCNT1* levels may be linked with elevated IgE levels; additional functional studies are warranted to explore the relationship between *GCNT1* and serum IgE concentration. Given that there is no previously published causal association between *GCNT1* and IgE concentration and that *GCNT1* appears to play a role in immune processes such as inflammatory Th1 homing, lymphocyte trafficking, and B cell differentiation, *GCNT1* represents a highly promising therapeutic target for the treatment and prevention of asthma and IgE-related diseases.

Two other nominally significant genes implicated in MR testing—*CCDC21* and *S100A13*—have no known mechanistic association with serum IgE concentration. *CCDC21* encodes a protein (centrosomal protein 85) that belongs to the centrosome-associated family of proteins. *S100A13* is a calcium binding gene that encodes for a protein (S100 calcium binding protein A13) belonging to the S100 family of proteins that are involved in a broad range of intracellular and extracellular functions. Extracellular S100 proteins often play crucial roles in regulating immune homeostasis and inflammation (32). By interacting with cell surface receptors such as RAGE (receptor for advanced glycation end products) in response to cell stress or inflammation, S100 proteins can activate intracellular signaling pathways that induce production of pro-inflammatory cytokines and lead to the migration of neutrophils, monocytes, and macrophages (32). Various extracellular S100 proteins have been associated with the pathogenesis of inflammatory diseases such as allergy. For example, multiple anti-allergic drugs such as amlexanox, cromolyn, and tranilast have been shown to bind S100A13 and block downstream RAGE signaling (32). While *CCDC21* and *S100A13* have not previously been shown to have roles in IgE regulation, our MR tests implicate them as potentially novel biomarkers or therapeutic targets.

In MR analyses of IgE-related diseases, we identified an IgE-associated gene expression signature that is “putatively” causal for asthma. Similar MR results for allergic diseases serve as further confirmation of our MR results of asthma. The identification of *GCNT1* as a causal gene for IgE concentration, asthma, and allergic diseases provides additional support for our hypothesis that IgE-associated gene expression changes impact IgE regulation and play a role in multiple IgE-related diseases. Based on our finding of a putatively causal role of *GCNT1* in IgE regulation and in asthma and allergic diseases, we hypothesize that *GCNT1* and the other IgE-associated genes identified in this study are related to the pathobiology of IgE-related diseases, including asthma and allergic diseases, and that they represent compelling therapeutic targets for treatment and prevention of these disorders.

Eosinophils drive IgE production. Eosinophil count is linked with IgE levels and was considered as residing in the causal pathway. Therefore, the primary analysis did not adjust for eosinophils. After adjusting eosinophils, the association of IgE with most genes is attenuated or disappears including the four putatively causal genes (*CLC, CCDC21, S100A13*, and *GCNT1*). This finding suggests that these eosinophil-linked genes may play a role in IgE production and IgE-related disorders such as asthma and allergy. After adjustment for eosinophil count, 12 genes remained significantly associated with IgE in the FHS cohort. Among the 12 genes, *ANXA1*, *IL5RA*, and *CD200R1* were replicated in the GACRS/CAMP cohorts. *IL5RA* also tested causal for asthma by MR. Strong correlations of *IL5RA* with eosinophils were observed in previous studies (33, 34). *IL5RA* regulates the development and function of eosinophils. Benralizumab, which targets *IL5RA*, is an approved drug to prevent eosinophilic and severe asthma. Further studies are needed to determine if there are other possible pathways to regulate IgE that are independent of eosinophil regulation.

There are several limitations to our study. First, we acknowledge that FHS transcriptomic data are restricted to expression in peripheral whole-blood derived RNA, which may not be representative of local tissue-specific effects. We did not use mucosal samples that are more relevant to IgE-related mucosal airway diseases. However, our study performed in blood can provide extensive information. Peripheral whole blood expression patterns can be linked to systemic inflammation and immune-related disorders including allergic diseases, in which IgE is involved, and may also reflect pathological changes occurring in other tissues, such as mucosa. For the four genes identified by MR analysis (*CLC*, *CCDC21*, *S100A13*, and *GCNT1*), we reviewed published literature and transcriptomic resources to check their transcriptional and translational (protein) properties in mucosal tissue. We found that *CLC* and *S100A13* were among the top genes showing differential expression in airway epithelium in asthma (35). Using data from the GTEx Portal (gtexportal.org/), we found that eQTLs for *GCNT1*, *CCDC21*, and *S100A13* are also identified in esophageal mucosal tissues, suggesting that genetic variants affect transcription of these genes in both blood and esophageal mucosal tissues. In addition, we checked expression levels of these four proteins in human bronchus (**Supplementary Figure 3**) and found that the staining intensity of *GCNT1* and *S100A13* was high in airway epithelial cells (36). In contrast, *CLC* and *CCDC21* showed low staining intensity. These results confirm the hypothesized relations between these genes and mucosal airway diseases, such as asthma. Further functional studies are needed to confirm and reveal the possible roles of these genes in the pathogenesis of mucosal airway diseases such as asthma. We further checked lung single cell data and found that *CCDC21*, *S100A13* and *GCNT1* show relatively high expression in macrophages in lung tissue (**Supplementary Table 8**), suggesting they may be involved in the immune defense of the airways.

Second, our study focused on total serum IgE, which measures the total amount of all forms of IgE antibodies in serum. A total IgE measurement does not show which specific forms of IgE are present. A history of specific allergic symptoms may elevate specific IgE against certain allergens and result in the marked increase in total IgE in serum (37). However, in some cases, markedly elevated total IgE (e.g. in widespread eczema) may result in weak positivity for specific IgE. Correlations between total and specific IgE were reported to be moderate in blood (38, 39). The relationships, including genetic background, between total serum IgE and IgE-related diseases are complex and need further investigation (40, 41).

Third, there are significant differences in mean age, IgE concentration, and percentages of participants with asthma in FHS compared to the GACRS/CAMP cohorts (**Table 1**). The average age of the FHS study participants was 55 years, which was significantly older than GACRS (9 years) and CAMP (20 years) participants. There was no significant difference in serum IgE concentration between the GACRS and CAMP cohorts, despite the ten-year age difference; however, the IgE levels of the GACRS and CAMP cohorts were considerably higher than those of FHS (log10 transformed IgE levels 2.5 kU/L and 2.5 kU/L vs. 1.52 kU/L). This is likely because all participants in GACRS and CAMP had asthma, which is associated with elevated IgE concentration. In contrast, only 7.6% of participants in FHS had asthma. Of the 216 IgE-associated transcripts (FDR<0.05) in FHS discovery, 59 genes bi-directionally replicated between the FHS and the GACRS/CAMP. This high degree of replication is notable given the previously described differences in cohort study populations.

Lastly, a limitation of this study is that gene expression was measured by array-based platforms. RNA sequencing outperforms array-based methods and can detect different isoforms of transcripts and is more sensitive for capturing low expressed transcripts.

Overall, many recent epigenome-wide association studies have been published that focus on the interactions of genetic, environment, and epigenetic factors underlying IgE, asthma, allergies, and other related traits (42–44). It is necessary to further investigate the correlation of environmental influences mediated by the epigenetic mechanisms contributing to IgE changes and IgE-related diseases, some of which may impact transcriptomic changes.

## Conclusion

We performed a TWAS of IgE and then probed the directional relations between IgE and gene expression, which identified four genes as causally associated with IgE levels. *CLC* is a well-documented gene with known associations with eosinophils and IgE; *CCDC21* and *S100A13* do not yet have well-understood associations with IgE and represent novel findings. Given its myriad of roles in the regulation of the immune response, *GCNT1* is a particularly attractive potential drug target given that in addition to its putatively causal relation to IgE levels it also was causal for asthma and allergic diseases. Our findings build upon prior knowledge of IgE regulation and provide a deeper understanding of the underlying molecular mechanisms. The IgE-associated genes that we identified—particularly those implicated in MR testing—can be explored as promising therapeutic targets for asthma and IgE-related diseases.

## Data availability statement

The data presented in the study are deposited in the dbGaP repository (http://www.ncbi.nlm.nih.gov/gap), accession number phs000007.

## Ethics statement

The studies involving human participants were reviewed and approved by Institutional Review Board at Boston University Medical Center, Boston, MA. The patients/participants provided their written informed consent to participate in this study.

## Author contributions

KR and TH wrote the manuscript. TH, S-JH, RK, and JL-S conducted the majority of analyses. All authors contributed to the article and approved the submitted version.
