# Supplementary material for: Transcriptome-wide association study of circulating IgE levels identifies novel targets for asthma and allergic diseases
Source: Front Immunol. 2023 Jan 30;14:1080071. doi: 10.3389/fimmu.2023.1080071 (PMC9922991; doi:10.3389/fimmu.2023.1080071)
Supplement: Supplementary file 1 [file DataSheet_1.docx]

Supplementary Material

**Supplementary Figure 1. Comparison of coefficient beta values of IgE-gene expression associations with and without cell type adjustment.** A) Without any cell type adjustment (X-axis) vs with cell type adjustment (Y-axis, cell types included white blood cells, red blood cells, lymphocytes, neutrophils, monocytes, platelets, and basophils, but did not include eosinophils). B) Without any cell type adjustment (X-axis) vs with cell type adjustment (Y-axis, cell types included white blood cells, red blood cells, lymphocytes, neutrophils, monocytes, platelets, basophils, and eosinophils). C) Adjusted whole blood cell types including eosinophils (X-axis) vs Adjusted other whole blood cell types but not including eosinophils.

**A B**

**
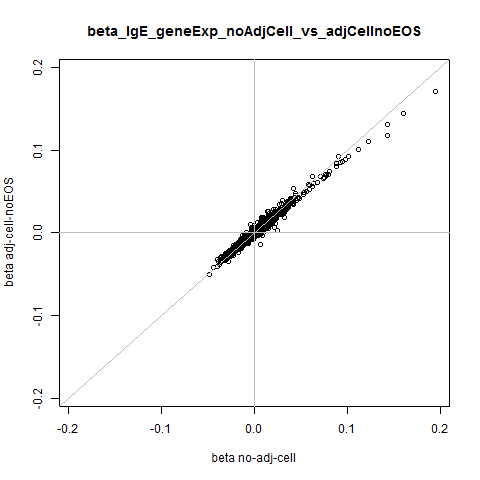
** **
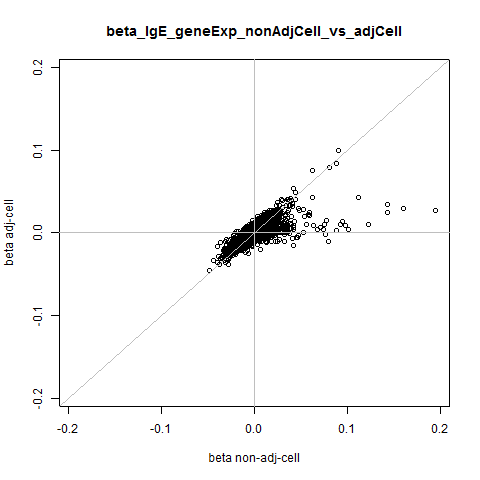
**

C

**
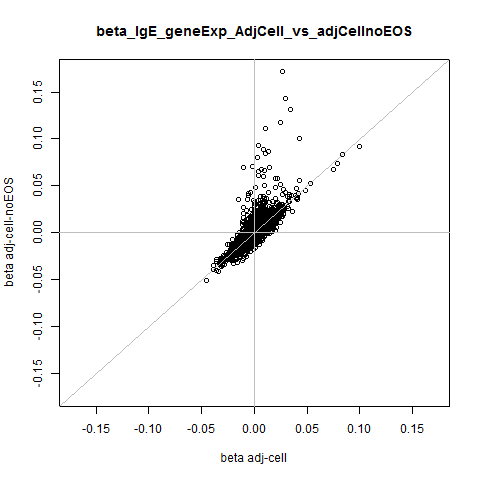
**

**
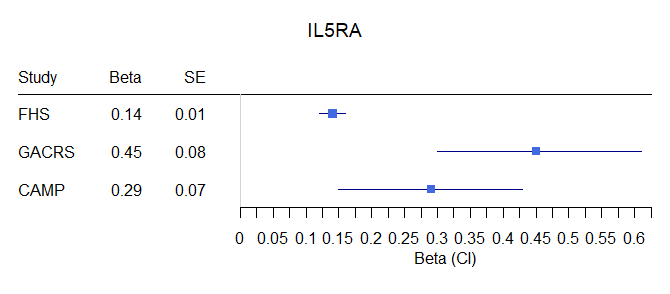
Supplementary Figure 2.** **Forest plots depicting beta value and 95% confidence interval (CI) of top 5 replicated genes on IgE in the FHS, GACRS and CAMP cohorts.** The forest plots used individual cohort data (i.e., non-meta-analyzed GACRS/CAMP data). The independent variable is normalized log-2 transformed gene expression value, and dependent variable is log transformed IgE concentration. Note: scales differ on each of the plots.

**
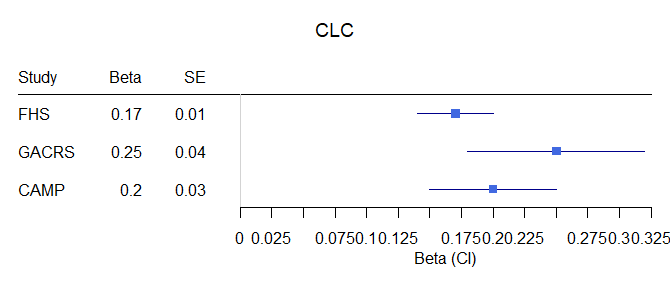

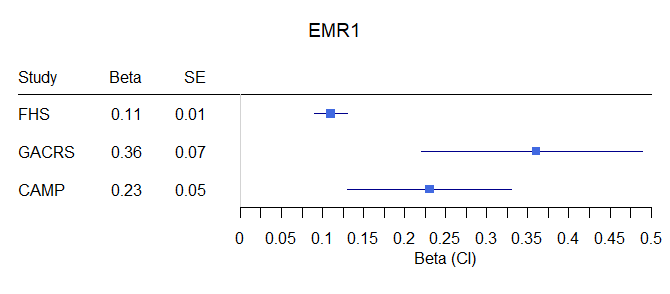
**

**
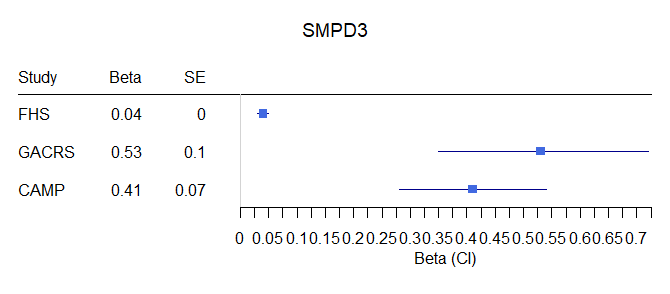

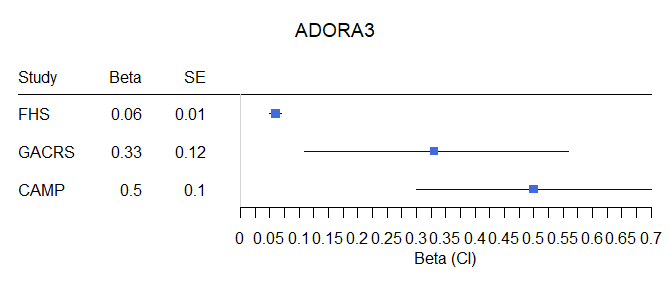
**

**Supplementary Figure 3. Immunochemical staining of human bronchus.**

**A B C**


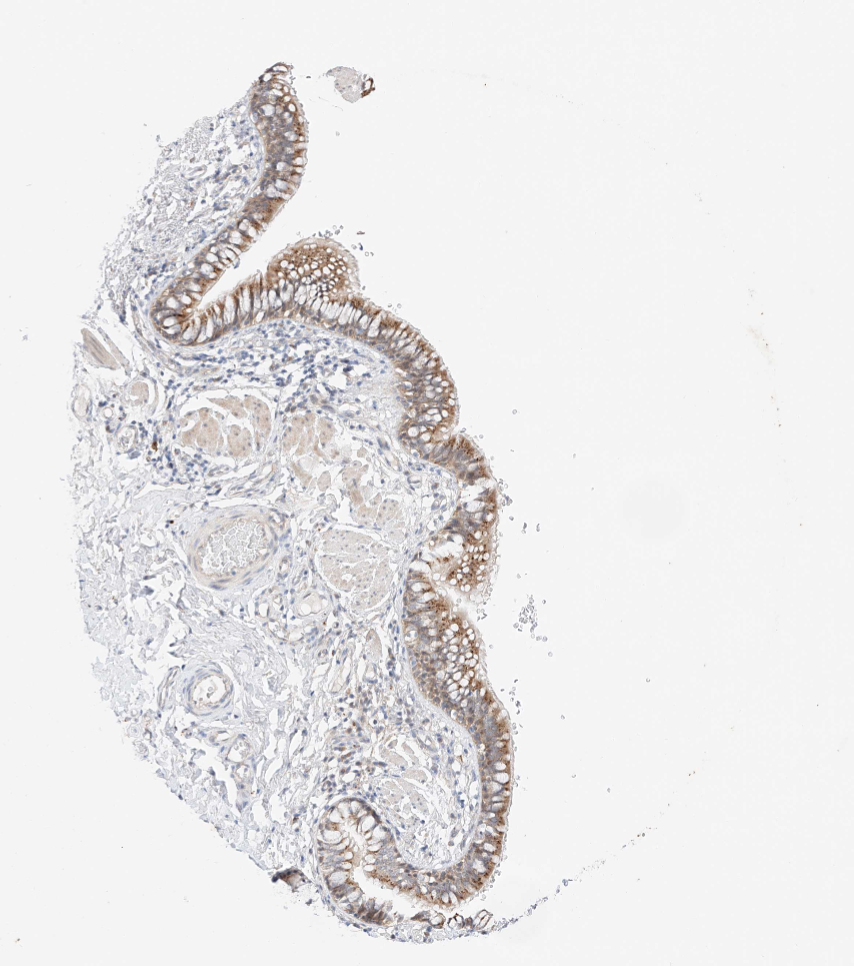

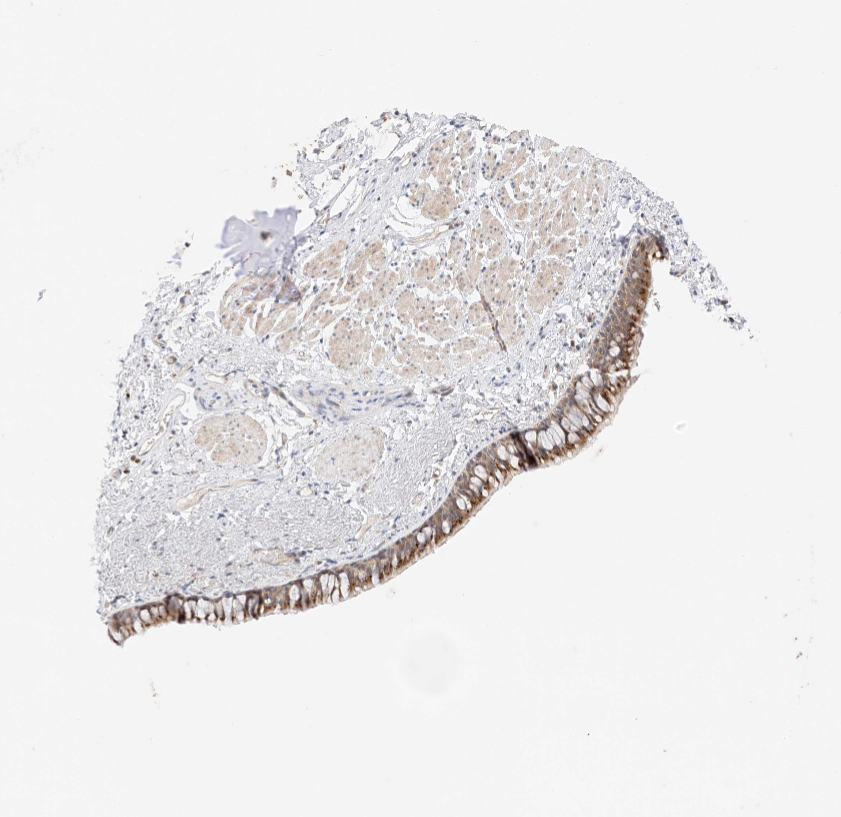

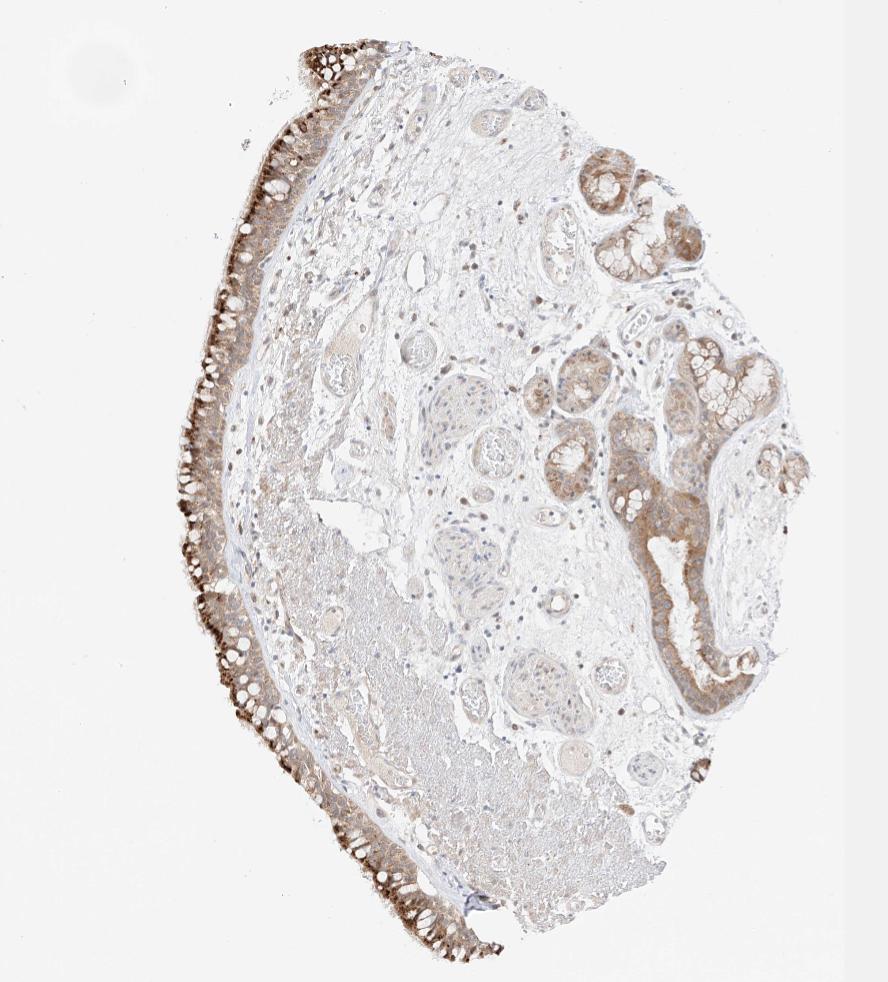


**D E**


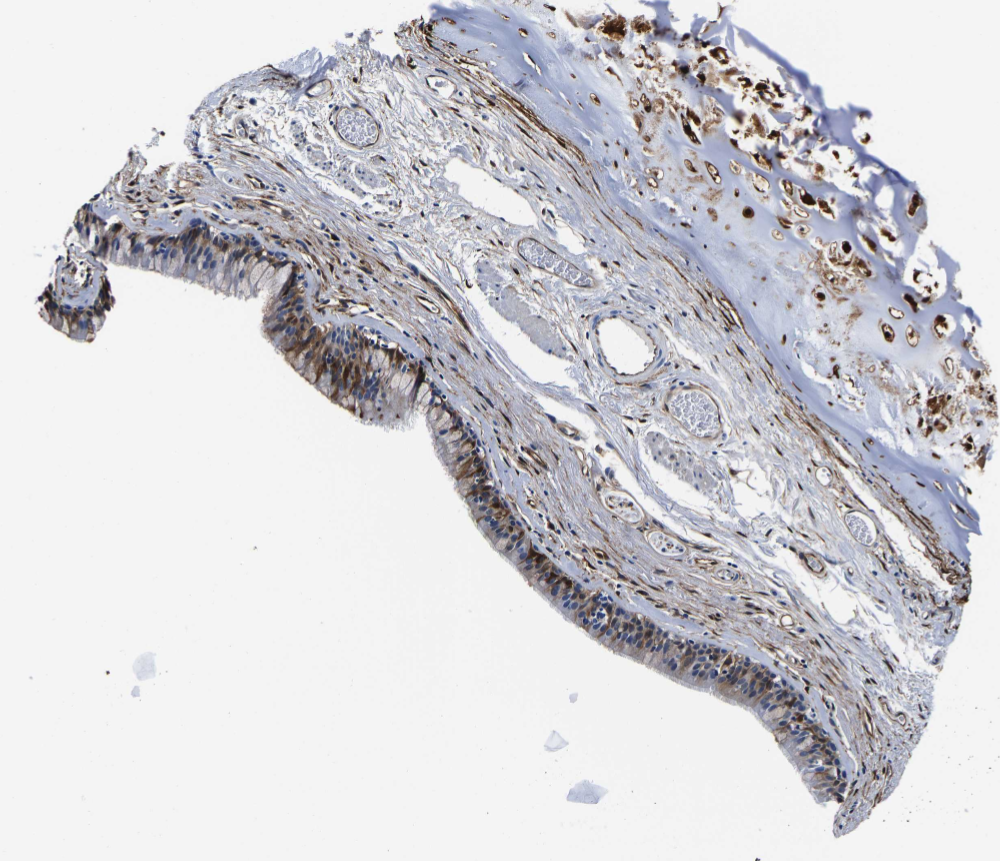

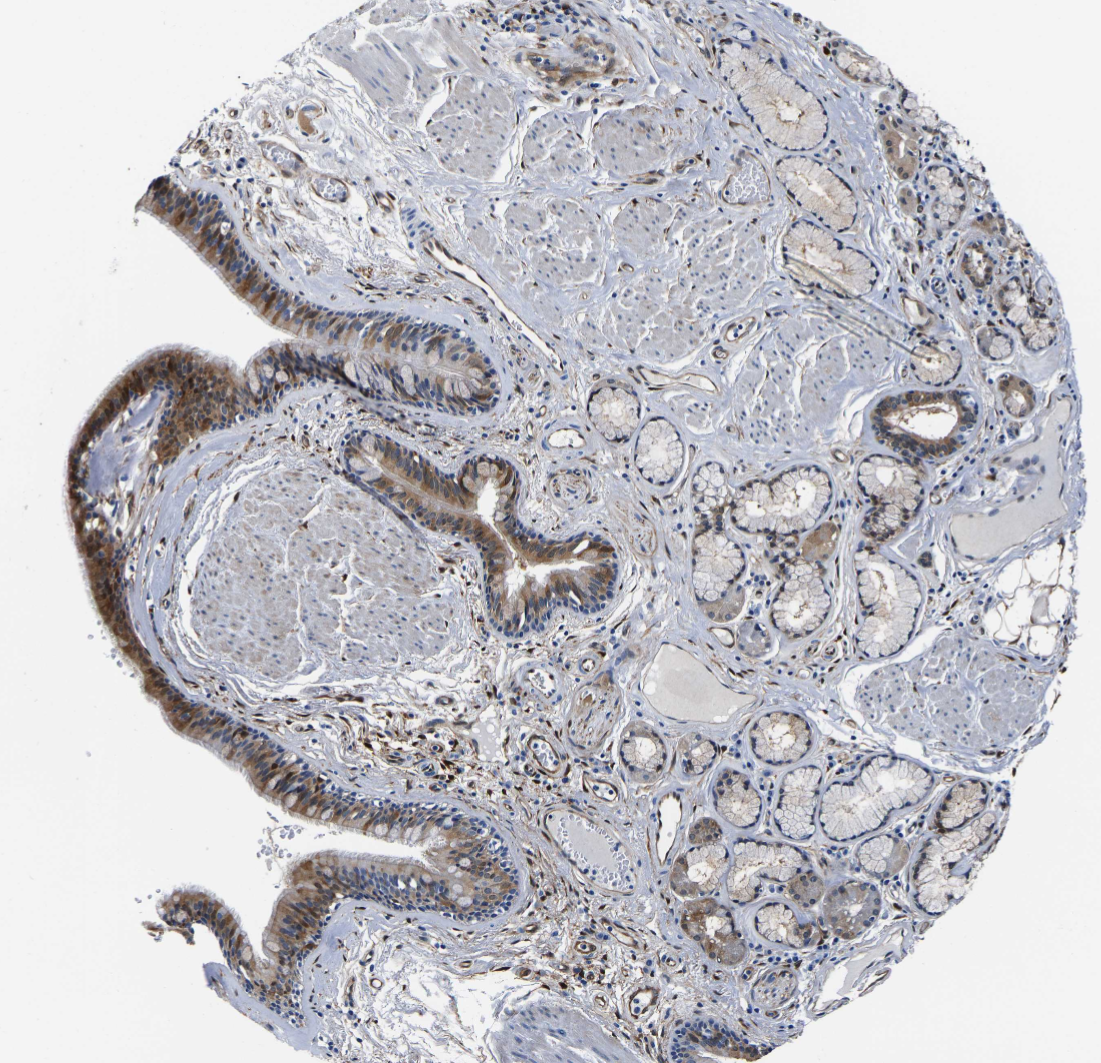


A)-C): GCNT1 (antibody: HPA031151); D)-E): S100A13 (antibody: HPA019592). A) Age 36, Female; B) Age 73, Female; C) Age 66, Male; D) Age 55, Female; E) Age 63, Female. Those images were downloaded from [Tissue expression of GCNT1 and S100A13 - Staining in bronchus - The Human Protein Atlas](https://www.proteinatlas.org/ENSG00000189171-S100A13/tissue/bronchus#img) (https://www.proteinatlas.org/). The staining intensity for CLC and CCDC21 is low (images of CLC and CCDC21 not included).

**Supplementary Table 1. Cell type proportions in the FHS participants.**

|  | Min. | 1st Qu. | Median | Mean | 3rd Qu. | Max. |
| --- | --- | --- | --- | --- | --- | --- |
| white blood cells | 0.8 | 5.2 | 6.0 | 6.1 | 6.9 | 16.8 |
| red blood cells | 3.4 | 4.3 | 4.5 | 4.5 | 4.8 | 6.2 |
| platelets | 114.2 | 225.2 | 248.9 | 249.8 | 273.8 | 453.7 |
| neutrophils | 27.8 | 54.2 | 59.0 | 59.1 | 63.9 | 96.1 |
| lymphocytes | 0.0 | 23.8 | 28.0 | 28.0 | 32.4 | 64.9 |
| monocytes | 2.9 | 7.6 | 8.8 | 8.9 | 10.1 | 24.4 |
| eosinophils | 0.0 | 2.0 | 2.9 | 3.2 | 4.0 | 16.0 |
| basophils | 0.0 | 0.6 | 0.8 | 0.8 | 0.9 | 2.3 |

The units for white blood cells and platelets are 10^3^ per uL, red blood cells are 10^6^ per uL, and the rest of the cells are measured in percentages (%).

**Supplementary Table 2.** Genes associated with total IgE levels in the FHS at FDR<0.05 (n=216).

| Gene Symbol | Chr | Beta | SE | P-Value | FDR Value |
| --- | --- | --- | --- | --- | --- |
| *IL5RA* | 3 | 0.144 | 0.011 | 1.88E-40 | 3.37E-36 |
| *SLC29A1* | 6 | 0.085 | 0.007 | 3.23E-34 | 2.88E-30 |
| *CLC* | 19 | 0.172 | 0.014 | 3.60E-32 | 2.15E-28 |
| *IL1RL1* | 2 | 0.131 | 0.011 | 6.41E-31 | 2.87E-27 |
| *EMR1* | 19 | 0.111 | 0.010 | 1.13E-28 | 4.04E-25 |
| *HRH4* | 18 | 0.118 | 0.011 | 8.13E-26 | 2.42E-22 |
| *DACH1* | 13 | 0.050 | 0.005 | 4.26E-25 | 1.09E-21 |
| *CCR4* | 3 | 0.084 | 0.008 | 1.04E-23 | 2.32E-20 |
| *TEC* | 4 | 0.067 | 0.007 | 1.31E-22 | 2.46E-19 |
| *SYNE1* | 6 | 0.068 | 0.007 | 1.38E-22 | 2.46E-19 |
| *ADORA3* | 1 | 0.061 | 0.006 | 1.32E-21 | 2.15E-18 |
| *ALOX15* | 17 | 0.093 | 0.010 | 7.64E-21 | 1.14E-17 |
| *CYSLTR2* | 13 | 0.089 | 0.010 | 4.43E-20 | 6.10E-17 |
| *SMPD3* | 16 | 0.039 | 0.004 | 5.92E-20 | 7.55E-17 |
| *IKZF2* | 2 | 0.058 | 0.007 | 3.18E-18 | 3.79E-15 |
| *PRSS33* | 16 | 0.028 | 0.003 | 5.30E-18 | 5.92E-15 |
| *PDE4D* | 5 | 0.037 | 0.004 | 7.99E-18 | 8.40E-15 |
| *CAT* | 11 | 0.066 | 0.008 | 4.86E-17 | 4.82E-14 |
| *SIGLEC8* | 19 | 0.042 | 0.005 | 1.52E-15 | 1.43E-12 |
| *IDO1* | 8 | 0.070 | 0.009 | 3.20E-15 | 2.86E-12 |
| *C2orf46* | 2 | 0.058 | 0.007 | 8.65E-15 | 7.37E-12 |
| *VSTM1* | 19 | 0.087 | 0.011 | 5.22E-14 | 4.24E-11 |
| *CD200R1* | 3 | 0.047 | 0.006 | 4.84E-13 | 3.76E-10 |
| *ARHGAP10* | 4 | 0.033 | 0.005 | 7.98E-13 | 5.94E-10 |
| *CCR3* | 3 | 0.070 | 0.010 | 2.38E-12 | 1.67E-09 |
| *CEBPE* | 14 | 0.048 | 0.007 | 2.43E-12 | 1.67E-09 |
| *GPR114* | 16 | 0.023 | 0.003 | 5.64E-12 | 3.73E-09 |
| *ANXA1* | 9 | 0.043 | 0.006 | 7.19E-12 | 4.59E-09 |
| *C15orf43* | 15 | 0.037 | 0.005 | 8.92E-12 | 5.50E-09 |
| *CAMK1* | 3 | 0.024 | 0.004 | 1.50E-11 | 8.95E-09 |
| *RNASE2* | 14 | 0.101 | 0.015 | 2.02E-11 | 1.16E-08 |
| *SEPT11* | 4 | 0.034 | 0.005 | 3.10E-11 | 1.73E-08 |
| *LGALS12* | 11 | 0.034 | 0.005 | 4.70E-11 | 2.54E-08 |
| *BHLHE40* | 3 | 0.037 | 0.006 | 6.79E-11 | 3.57E-08 |
| *ESYT1* | 12 | 0.030 | 0.005 | 1.40E-10 | 7.13E-08 |
| *OLIG2* | 21 | 0.028 | 0.004 | 1.53E-10 | 7.58E-08 |
| *CSF1* | 1 | 0.023 | 0.004 | 2.35E-10 | 1.14E-07 |
| *C6orf97* | 6 | 0.032 | 0.005 | 2.53E-10 | 1.19E-07 |
| *PLAC4* | 21 | 0.039 | 0.006 | 3.04E-10 | 1.39E-07 |
| *CD9* | 12 | 0.048 | 0.008 | 4.27E-10 | 1.91E-07 |
| *P2RY14* | 3 | 0.070 | 0.011 | 5.18E-10 | 2.26E-07 |
| *EEF2K* | 16 | 0.021 | 0.003 | 6.26E-10 | 2.66E-07 |
| *SRGAP3* | 3 | 0.015 | 0.003 | 1.25E-09 | 5.07E-07 |
| *FGFR2* | 10 | 0.031 | 0.005 | 1.22E-09 | 5.07E-07 |
| *INPP1* | 2 | 0.032 | 0.005 | 1.28E-09 | 5.08E-07 |
| *CCL23* | 17 | 0.039 | 0.007 | 2.13E-09 | 8.26E-07 |
| *C6orf114* | 6 | 0.038 | 0.006 | 3.31E-09 | 1.23E-06 |
| *TRERF1* | 6 | 0.016 | 0.003 | 3.22E-09 | 1.23E-06 |
| *UGT2B28* | 4 | 0.080 | 0.014 | 3.83E-09 | 1.40E-06 |
| *DOK2* | 8 | 0.025 | 0.004 | 6.82E-09 | 2.44E-06 |
| *SAMSN1* | 21 | 0.031 | 0.005 | 1.06E-08 | 3.70E-06 |
| *CD24* | Y | 0.060 | 0.011 | 1.65E-08 | 5.68E-06 |
| *FBP1* | 9 | 0.025 | 0.005 | 2.08E-08 | 7.01E-06 |
| *IL2RA* | 10 | 0.056 | 0.010 | 2.80E-08 | 9.26E-06 |
| *PGD* | 1 | 0.026 | 0.005 | 3.01E-08 | 9.79E-06 |
| *FRY* | 13 | 0.025 | 0.004 | 3.77E-08 | 1.20E-05 |
| *CACNA1D* | 3 | 0.014 | 0.003 | 4.86E-08 | 1.46E-05 |
| *GATA3* | 10 | 0.024 | 0.004 | 4.91E-08 | 1.46E-05 |
| *SLC4A8* | 12 | 0.022 | 0.004 | 4.91E-08 | 1.46E-05 |
| *PNPLA6* | 19 | 0.012 | 0.002 | 4.81E-08 | 1.46E-05 |
| *GAPT* | 5 | 0.036 | 0.007 | 5.16E-08 | 1.51E-05 |
| *FANK1* | 10 | 0.034 | 0.006 | 5.81E-08 | 1.67E-05 |
| *CCDC86* | 11 | 0.022 | 0.004 | 6.15E-08 | 1.74E-05 |
| *ACSM1* | 16 | 0.023 | 0.004 | 7.36E-08 | 2.06E-05 |
| *RAB44* | 6 | 0.024 | 0.004 | 8.85E-08 | 2.43E-05 |
| *CDK15* | 2 | 0.021 | 0.004 | 9.16E-08 | 2.48E-05 |
| *SLC16A14* | 2 | 0.026 | 0.005 | 1.03E-07 | 2.74E-05 |
| *ARHGEF6* | X | 0.022 | 0.004 | 1.45E-07 | 3.80E-05 |
| *SIGLEC10* | 19 | 0.033 | 0.006 | 1.55E-07 | 4.01E-05 |
| *GCNT1* | 9 | 0.028 | 0.005 | 1.67E-07 | 4.25E-05 |
| *SMAD5* | 5 | 0.028 | 0.005 | 1.81E-07 | 4.56E-05 |
| *DSC2* | 18 | 0.052 | 0.010 | 3.02E-07 | 7.50E-05 |
| *INPP5B* | 1 | 0.026 | 0.005 | 3.82E-07 | 9.35E-05 |
| *HCRP1* | 6 | 0.035 | 0.007 | 4.34E-07 | 1.05E-04 |
| *NDFIP2* | 13 | 0.022 | 0.004 | 4.81E-07 | 1.15E-04 |
| *TNIK* | 3 | 0.024 | 0.005 | 6.02E-07 | 1.42E-04 |
| *CASP3* | 4 | 0.028 | 0.006 | 6.64E-07 | 1.54E-04 |
| *CD101* | 1 | 0.030 | 0.006 | 9.35E-07 | 2.14E-04 |
| *RPS6KA2* | 6 | 0.015 | 0.003 | 9.55E-07 | 2.15E-04 |
| *BACE2* | 21 | 0.027 | 0.006 | 9.61E-07 | 2.15E-04 |
| *GPR44* | 11 | 0.031 | 0.006 | 1.21E-06 | 2.66E-04 |
| *SEMA5A* | 5 | 0.012 | 0.002 | 1.29E-06 | 2.81E-04 |
| *P2RY2* | 11 | 0.021 | 0.004 | 1.39E-06 | 2.99E-04 |
| *THBS4* | 5 | 0.013 | 0.003 | 1.59E-06 | 3.39E-04 |
| *GPI* | 19 | 0.022 | 0.005 | 1.79E-06 | 3.77E-04 |
| *ZMYND11* | 10 | 0.020 | 0.004 | 1.91E-06 | 3.97E-04 |
| *HRASLS5* | 11 | 0.021 | 0.004 | 2.02E-06 | 4.15E-04 |
| *EPN2* | 17 | 0.013 | 0.003 | 2.31E-06 | 4.69E-04 |
| *LPCAT2* | 16 | 0.025 | 0.005 | 2.51E-06 | 5.05E-04 |
| *FBN1* | 15 | 0.010 | 0.002 | 2.62E-06 | 5.21E-04 |
| *GRB10* | 7 | 0.015 | 0.003 | 2.71E-06 | 5.32E-04 |
| *OLIG1* | 21 | 0.016 | 0.003 | 3.03E-06 | 5.89E-04 |
| *ID2* | 2 | 0.043 | 0.009 | 3.32E-06 | 6.36E-04 |
| *ABTB2* | 11 | 0.013 | 0.003 | 3.34E-06 | 6.36E-04 |
| *ALS2* | 2 | 0.017 | 0.004 | 3.38E-06 | 6.36E-04 |
| *INADL* | 1 | 0.021 | 0.004 | 3.48E-06 | 6.48E-04 |
| *TUBGCP4* | 15 | 0.021 | 0.005 | 3.85E-06 | 7.09E-04 |
| *HIF1A* | 14 | 0.024 | 0.005 | 6.14E-06 | 1.11E-03 |
| *ABCC1* | 16 | 0.015 | 0.003 | 6.09E-06 | 1.11E-03 |
| *VLDLR* | 9 | 0.027 | 0.006 | 6.71E-06 | 1.20E-03 |
| *PMP22* | 17 | 0.029 | 0.006 | 6.86E-06 | 1.21E-03 |
| *RGS1* | 1 | 0.032 | 0.007 | 6.97E-06 | 1.22E-03 |
| *S100A10* | 1 | 0.030 | 0.007 | 7.27E-06 | 1.26E-03 |
| *PLIN2* | 9 | 0.028 | 0.006 | 8.11E-06 | 1.39E-03 |
| *PTPN22* | 1 | 0.021 | 0.005 | 8.45E-06 | 1.44E-03 |
| *ACACB* | 12 | 0.012 | 0.003 | 9.30E-06 | 1.57E-03 |
| *ADAM19* | 5 | 0.017 | 0.004 | 9.89E-06 | 1.63E-03 |
| *DAPK1* | 9 | 0.027 | 0.006 | 9.93E-06 | 1.63E-03 |
| *AKR1C3* | 10 | 0.042 | 0.009 | 9.79E-06 | 1.63E-03 |
| *CD244* | 1 | 0.022 | 0.005 | 1.29E-05 | 2.09E-03 |
| *PI16* | 6 | 0.022 | 0.005 | 1.31E-05 | 2.11E-03 |
| *HES1* | 3 | 0.014 | 0.003 | 1.38E-05 | 2.21E-03 |
| *UBXN2A* | 2 | -0.031 | 0.007 | 1.42E-05 | 2.25E-03 |
| *GALC* | 14 | 0.016 | 0.004 | 1.52E-05 | 2.38E-03 |
| *ITGAM* | 16 | 0.018 | 0.004 | 1.54E-05 | 2.40E-03 |
| *FAM124B* | 2 | 0.020 | 0.005 | 1.56E-05 | 2.41E-03 |
| *CYP4F12* | 19 | 0.030 | 0.007 | 1.61E-05 | 2.47E-03 |
| *WEE1* | 11 | 0.020 | 0.005 | 2.13E-05 | 3.22E-03 |
| *CPT1A* | 11 | 0.024 | 0.006 | 2.33E-05 | 3.50E-03 |
| *AHR* | 7 | 0.022 | 0.005 | 2.38E-05 | 3.54E-03 |
| *NCKAP1L* | 12 | 0.014 | 0.003 | 3.01E-05 | 4.44E-03 |
| *CLINT1* | 5 | 0.015 | 0.004 | 3.09E-05 | 4.53E-03 |
| *PAPSS1* | 4 | 0.030 | 0.007 | 3.41E-05 | 4.95E-03 |
| *KIT* | 4 | 0.018 | 0.004 | 3.68E-05 | 5.31E-03 |
| *ATP8B2* | 1 | 0.016 | 0.004 | 4.69E-05 | 6.66E-03 |
| *S100Z* | 5 | 0.025 | 0.006 | 4.70E-05 | 6.66E-03 |
| *SLC24A3* | 20 | 0.015 | 0.004 | 4.74E-05 | 6.67E-03 |
| *SYCP2L* | 6 | 0.031 | 0.008 | 4.83E-05 | 6.75E-03 |
| *KLRF1* | 12 | 0.053 | 0.013 | 4.90E-05 | 6.79E-03 |
| *CD97* | 19 | 0.018 | 0.004 | 5.11E-05 | 7.02E-03 |
| *CMAH* | 6 | 0.020 | 0.005 | 5.17E-05 | 7.05E-03 |
| *DRAM2* | 1 | -0.020 | 0.005 | 5.96E-05 | 8.07E-03 |
| *SLFN13* | 17 | 0.027 | 0.007 | 7.38E-05 | 9.91E-03 |
| *TTC38* | 22 | 0.015 | 0.004 | 7.90E-05 | 1.05E-02 |
| *KLF6* | 10 | 0.017 | 0.004 | 8.37E-05 | 1.10E-02 |
| *EBP* | X | 0.021 | 0.005 | 8.34E-05 | 1.10E-02 |
| *CAMK1D* | 10 | 0.023 | 0.006 | 8.83E-05 | 1.15E-02 |
| *HCG4* | 6 | -0.022 | 0.006 | 9.03E-05 | 1.17E-02 |
| *IL17RB* | 3 | 0.013 | 0.003 | 9.20E-05 | 1.18E-02 |
| *FCRL2* | 1 | -0.031 | 0.008 | 1.00E-04 | 1.28E-02 |
| *SLC35D1* | 1 | 0.023 | 0.006 | 1.01E-04 | 1.28E-02 |
| *GFI1B* | 9 | 0.017 | 0.004 | 1.23E-04 | 1.55E-02 |
| *MLKL* | 16 | 0.024 | 0.006 | 1.27E-04 | 1.59E-02 |
| *STK17A* | 7 | -0.015 | 0.004 | 1.32E-04 | 1.63E-02 |
| *CLDN2* | X | -0.015 | 0.004 | 1.31E-04 | 1.63E-02 |
| *TRIB1* | 8 | 0.017 | 0.005 | 1.35E-04 | 1.64E-02 |
| *HEATR5A* | 14 | 0.040 | 0.010 | 1.35E-04 | 1.64E-02 |
| *CD63* | 12 | 0.013 | 0.003 | 1.36E-04 | 1.64E-02 |
| *SUSD1* | 9 | 0.016 | 0.004 | 1.45E-04 | 1.72E-02 |
| *NUPL1* | 13 | -0.012 | 0.003 | 1.44E-04 | 1.72E-02 |
| *NUP93* | 16 | 0.017 | 0.004 | 1.44E-04 | 1.72E-02 |
| *RACGAP1* | 12 | 0.014 | 0.004 | 1.49E-04 | 1.76E-02 |
| *CD52* | 1 | 0.021 | 0.005 | 1.52E-04 | 1.77E-02 |
| *SEMA7A* | 15 | 0.012 | 0.003 | 1.58E-04 | 1.83E-02 |
| *GSTM4* | 1 | 0.043 | 0.011 | 1.61E-04 | 1.84E-02 |
| *ORM2* | 9 | -0.036 | 0.009 | 1.60E-04 | 1.84E-02 |
| *IGJ* | 4 | 0.074 | 0.020 | 1.64E-04 | 1.87E-02 |
| *CRIP1* | 14 | 0.041 | 0.011 | 1.68E-04 | 1.90E-02 |
| *FCRLA* | 1 | -0.021 | 0.006 | 1.71E-04 | 1.91E-02 |
| *SYNE2* | 14 | 0.021 | 0.006 | 1.70E-04 | 1.91E-02 |
| *PLD3* | 19 | 0.011 | 0.003 | 1.72E-04 | 1.91E-02 |
| *KLHL6* | 3 | 0.018 | 0.005 | 1.73E-04 | 1.91E-02 |
| *GZMB* | 14 | 0.040 | 0.011 | 1.75E-04 | 1.92E-02 |
| *S100A13* | 1 | -0.039 | 0.010 | 1.76E-04 | 1.92E-02 |
| *ALS2CR12* | 2 | -0.030 | 0.008 | 1.79E-04 | 1.93E-02 |
| *ACSL4* | X | 0.017 | 0.004 | 1.79E-04 | 1.93E-02 |
| *HRASLS2* | 11 | 0.029 | 0.008 | 1.81E-04 | 1.94E-02 |
| *RGS6* | 14 | -0.021 | 0.005 | 1.91E-04 | 2.02E-02 |
| *PRPS1* | X | 0.023 | 0.006 | 1.91E-04 | 2.02E-02 |
| *BRI3BP* | 12 | 0.014 | 0.004 | 1.94E-04 | 2.04E-02 |
| *DIXDC1* | 11 | 0.014 | 0.004 | 2.12E-04 | 2.22E-02 |
| *GPR137B* | 1 | 0.024 | 0.007 | 2.15E-04 | 2.23E-02 |
| *PDE8A* | 15 | 0.014 | 0.004 | 2.26E-04 | 2.33E-02 |
| *INPP5A* | 10 | 0.015 | 0.004 | 2.30E-04 | 2.36E-02 |
| *SCPEP1* | 17 | 0.017 | 0.005 | 2.31E-04 | 2.36E-02 |
| *PRF1* | 10 | 0.028 | 0.008 | 2.40E-04 | 2.44E-02 |
| *CACNG6* | 19 | 0.013 | 0.004 | 2.43E-04 | 2.46E-02 |
| *CLCNKB* | 1 | 0.014 | 0.004 | 2.55E-04 | 2.56E-02 |
| *COBLL1* | 2 | -0.019 | 0.005 | 2.66E-04 | 2.64E-02 |
| *SMARCA1* | X | -0.014 | 0.004 | 2.65E-04 | 2.64E-02 |
| *ZNF610* | 19 | -0.021 | 0.006 | 2.73E-04 | 2.69E-02 |
| *SMARCC2* | 12 | 0.014 | 0.004 | 2.82E-04 | 2.77E-02 |
| *P4HA1* | 10 | 0.016 | 0.005 | 2.92E-04 | 2.85E-02 |
| *DZIP3* | 3 | -0.021 | 0.006 | 3.05E-04 | 2.96E-02 |
| *PPM1L* | 3 | 0.026 | 0.007 | 3.09E-04 | 2.99E-02 |
| *NELL2* | 12 | -0.031 | 0.009 | 3.14E-04 | 3.02E-02 |
| *SLC7A8* | 14 | 0.017 | 0.005 | 3.33E-04 | 3.18E-02 |
| *CCDC21* | 1 | 0.015 | 0.004 | 3.46E-04 | 3.29E-02 |
| *ITGB1* | 10 | 0.018 | 0.005 | 3.56E-04 | 3.37E-02 |
| *ACVR1B* | 12 | 0.016 | 0.004 | 3.79E-04 | 3.56E-02 |
| *TARBP1* | 1 | -0.012 | 0.003 | 3.84E-04 | 3.57E-02 |
| *CAMKK2* | 12 | 0.013 | 0.004 | 3.85E-04 | 3.57E-02 |
| *PARP4* | 13 | 0.015 | 0.004 | 3.84E-04 | 3.57E-02 |
| *RTKN2* | 10 | 0.024 | 0.007 | 3.95E-04 | 3.64E-02 |
| *SPNS3* | 17 | 0.009 | 0.003 | 4.12E-04 | 3.76E-02 |
| *GPR34* | X | 0.025 | 0.007 | 4.11E-04 | 3.76E-02 |
| *GAS2L3* | 12 | -0.026 | 0.007 | 4.19E-04 | 3.80E-02 |
| *LRRCC1* | 8 | -0.021 | 0.006 | 4.27E-04 | 3.86E-02 |
| *C1orf162* | 1 | 0.018 | 0.005 | 4.30E-04 | 3.87E-02 |
| *ARL6IP6* | 2 | 0.021 | 0.006 | 4.65E-04 | 4.13E-02 |
| *THUMPD1* | 16 | 0.018 | 0.005 | 4.64E-04 | 4.13E-02 |
| *PDK4* | 7 | 0.027 | 0.008 | 4.70E-04 | 4.16E-02 |
| *NENF* | 1 | -0.018 | 0.005 | 4.84E-04 | 4.18E-02 |
| *VKORC1L1* | 7 | 0.018 | 0.005 | 4.78E-04 | 4.18E-02 |
| *C3AR1* | 12 | 0.034 | 0.010 | 4.81E-04 | 4.18E-02 |
| *SELPLG* | 12 | 0.015 | 0.004 | 4.82E-04 | 4.18E-02 |
| *PSMF1* | 20 | -0.031 | 0.009 | 4.77E-04 | 4.18E-02 |
| *TAB2* | 6 | -0.014 | 0.004 | 4.89E-04 | 4.21E-02 |
| *ANGPTL1* | 1 | -0.028 | 0.008 | 5.06E-04 | 4.33E-02 |
| *TNFRSF9* | 1 | -0.024 | 0.007 | 5.15E-04 | 4.38E-02 |
| *TLE1* | 9 | 0.016 | 0.005 | 5.26E-04 | 4.46E-02 |
| *FMNL3* | 12 | 0.011 | 0.003 | 5.31E-04 | 4.47E-02 |
| *DEPDC5* | 22 | 0.011 | 0.003 | 5.35E-04 | 4.49E-02 |
| *RAB5A* | 3 | -0.016 | 0.005 | 5.53E-04 | 4.62E-02 |
| *C9orf91* | 9 | -0.014 | 0.004 | 5.56E-04 | 4.62E-02 |
| *WDR7* | 18 | 0.011 | 0.003 | 5.84E-04 | 4.83E-02 |

**Supplementary Table 3.** Top 12 genes associated with IgE levels in the FHS cohort (after adjustment for eosinophil count). The 6 genes that pass Bonferroni-correction are in bold.

| Transcript Number | Gene Symbol | Chr | P-Value | FDR Value |
| --- | --- | --- | --- | --- |
| 2616131 | ***CCR4*** | 3 | 4.99E-23 | 8.91E-19 |
| 3234277 | ***GATA3*** | 10 | 2.33E-09 | 2.08E-05 |
| 3269694 | ***FANK1*** | 10 | 1.47E-07 | 8.75E-04 |
| 2847967 | ***SEMA5A*** | 5 | 1.93E-06 | 7.10E-03 |
| 2905296 | ***PI16*** | 6 | 1.99E-06 | 7.10E-03 |
| 3174816 | ***ANXA1*** | 9 | 2.53E-06 | 7.54E-03 |
| 2660617 | *IL5RA* | 3 | 5.97E-06 | 1.52E-02 |
| 3352948 | *SORL1* | 11 | 8.68E-06 | 1.94E-02 |
| 2688955 | *CD200R1* | 3 | 1.34E-05 | 2.40E-02 |
| 3233049 | *AKR1C3* | 10 | 1.34E-05 | 2.40E-02 |
| 2884578 | *CCNJL* | 5 | 2.33E-05 | 3.48E-02 |
| 3275729 | *IL2RA* | 10 | 2.19E-05 | 3.48E-02 |

**Supplementary Table 4.** List of replicated gene transcripts (n=114) associated with circulating IgE levels between meta-analyzed replication cohorts and FHS. The * denotes genes that are linked to two separate transcripts.

| Gene Symbol | Gene Name | GACRS/CAMP FDR Value | GACRS/CAMP P-Value | FHS P-Value |
| --- | --- | --- | --- | --- |
| *OLIG2* | Oligodendrocyte Transcription Factor 2 | 5.42E-25 | 4.34E-29 | 1.53E-10 |
| *TFF3* | Trefoil Factor 3 | 5.42E-25 | 3.46E-29 | 3.42E-02 |
| *CEBPE* | CCAAT Enhancer Binding Protein Epsilon | 1.65E-24 | 1.99E-28 | 2.43E-12 |
| *PRSS33* | Serine Protease 33 | 1.57E-23 | 2.51E-27 | 5.30E-18 |
| *CCL23* | C‐C Motif Chemokine Ligand 23 | 4.82E-21 | 9.65E-25 | 2.13E-09 |
| *CLC* | Charcot‐Leyden Crystal Galectin | 2.08E-20 | 5.00E-24 | 3.60E-32 |
| *SIGLEC8* | Sialic Acid Binding Ig-Like Lectin 8 | 2.96E-18 | 1.07E-21 | 1.52E-15 |
| *GPR44* | Prostaglandin D2 Receptor 2 | 3.23E-18 | 1.29E-21 | 1.21E-06 |
| *OLIG1* | Oligodendrocyte Transcription Factor 1 | 4.26E-17 | 1.88E-20 | 3.03E-06 |
| *IDO1* | Indoleamine 2,3‐Dioxygenase 1 | 9.81E-16 | 5.50E-19 | 3.20E-15 |
| *SPNS3* | Sphingolipid Transporter 3 (Putative) | 4.59E-15 | 2.76E-18 | 4.12E-04 |
| *SMPD3* | Sphingomyelin Phosphodiesterase 3 | 2.96E-13 | 2.14E-16 | 5.92E-20 |
| *VSTM1* | V‐Set & Transmembrane Domain Containing 1 | 1.73E-12 | 1.32E-15 | 5.22E-14 |
| *CCR3* | C‐C motif Chemokine Receptor 3 | 8.90E-12 | 7.13E-15 | 2.38E-12 |
| *LGALS12* | Galectin 12 | 9.77E-12 | 8.22E-15 | 4.70E-11 |
| *INPP1* | Inositol Polyphosphate‐1‐Phosphatase | 3.95E-11 | 3.49E-14 | 1.28E-09 |
| *RNF14* | Ring Finger Protein 14 | 3.95E-11 | 3.64E-14 | 8.39E-02 |
| *CAMK1* | Calcium/Calmodulin Dependent Protein Kinase I | 5.31E-11 | 5.10E-14 | 1.50E-11 |
| *LTC4S* | Leukotriene C4 Synthase | 1.07E-10 | 1.07E-13 | 4.39E-01 |
| *GFOD1* | Glucose-Fructose Oxidoreductase Domain Containing 1 | 2.21E-10 | 2.30E-13 | 1.13E-01 |
| *RHOBTB3* | Rho Related BTB Domain Containing 3 | 2.71E-10 | 2.94E-13 | 1.90E-02 |
| *CYP4F12* | Cytochrome P450 Family 4 Subfamily F Member 12 | 2.91E-10 | 3.26E-13 | 1.61E-05 |
| *ACOT11** | Acyl-CoA Thioesterase 11 | 3.28E-10 | 3.81E-13 | 9.94E-01 |
| *ACOT11** | Acyl-CoA Thioesterase 11 | 3.28E-10 | 3.81E-13 | 7.68E-04 |
| *PSTPIP2* | Proline-Serine-Threonine Phosphatase Interacting Protein 2 | 3.23E-09 | 3.88E-12 | 2.37E-02 |
| *IL5RA* | Interleukin 5 Receptor Subunit Alpha | 6.51E-09 | 8.08E-12 | 1.88E-40 |
| *MYB* | MYB Proto-Oncogene, Transcription Factor | 9.53E-09 | 1.22E-11 | 1.93E-02 |
| *EMR1* | Adhesion G Protein‐Coupled Receptor E1 (ADGRE1) | 1.34E-08 | 1.89E-11 | 1.13E-28 |
| *OXER1* | Oxoeicosanoid Receptor 1 | 1.34E-08 | 1.89E-11 | 2.54E-02 |
| *THBS4* | Thrombospondin 4 | 1.34E-08 | 1.93E-11 | 1.59E-06 |
| *EEF2K* | Eukaryotic Elongation Factor 2 Kinase | 1.73E-08 | 2.57E-11 | 6.26E-10 |
| *COL9A2* | Collagen Type IX Alpha 2 Chain | 1.31E-07 | 1.99E-10 | 1.33E-01 |
| *SRGAP3* | SLIT-ROBO Rho GTPase Activating Protein 3 | 1.44E-06 | 2.24E-09 | 1.25E-09 |
| *RNASE3* | Ribonuclease A Family Member 3 | 1.63E-06 | 2.61E-09 | 4.35E-02 |
| *BACE2* | Beta-Secretase 2 | 1.81E-06 | 2.96E-09 | 9.61E-07 |
| *FAM124B* | Family With Sequence Similarity 124 Member B | 1.91E-06 | 3.22E-09 | 1.56E-05 |
| *EPAS1* | Endothelial PAS Domain Protein 1 | 1.94E-06 | 3.33E-09 | 7.58E-04 |
| *C13orf27* | Testis Expressed 30 | 2.17E-06 | 3.82E-09 | 2.55E-03 |
| *RNASE2* | Ribonuclease A Family Member 2 | 2.48E-06 | 4.47E-09 | 2.02E-11 |
| *HYAL3* | Hyaluronidase 3 | 6.50E-06 | 1.20E-08 | 2.83E-01 |
| *ACACB* | Acetyl-CoA Carboxylase Beta | 7.50E-06 | 1.41E-08 | 9.30E-06 |
| *GPR114* | Adhesion G Protein-Coupled Receptor G5 | 8.39E-06 | 1.61E-08 | 5.64E-12 |
| *ADORA3* | Adenosine A3 Receptor | 1.03E-05 | 2.02E-08 | 1.32E-21 |
| *TRERF1** | Transcriptional Regulating Factor 1 | 2.71E-05 | 5.43E-08 | 1.62E-02 |
| *TRERF1** | Transcriptional Regulating Factor 1 | 2.71E-05 | 5.43E-08 | 3.22E-09 |
| *HSD3B7* | Hydroxy-Delta-5-Steroid Dehydrogenase, 3 Beta- And Steroid Delta-Isomerase 7 | 2.75E-05 | 5.62E-08 | 2.83E-01 |
| *SIGLEC10* | Sialic Acid Binding Ig Like Lectin 10 | 3.27E-05 | 6.95E-08 | 1.55E-07 |
| *FBP1* | Fructose-Bisphosphatase 1 | 3.52E-05 | 7.75E-08 | 2.08E-08 |
| *SLC16A14* | Solute Carrier Family 16 Member 14 | 3.52E-05 | 7.63E-08 | 1.03E-07 |
| *ATP8B3* | ATPase Phospholipid Transporting 8B3 | 6.39E-05 | 1.43E-07 | 5.29E-01 |
| *ACSF2* | Acyl-CoA Synthetase Family Member 2 | 1.43E-04 | 3.27E-07 | 5.77E-02 |
| *CAT* | Catalase | 1.94E-04 | 4.50E-07 | 4.86E-17 |
| *HES4* | Hes Family BHLH Transcription Factor 4 | 2.39E-04 | 5.66E-07 | 3.65E-02 |
| *SSH3* | Slingshot Protein Phosphatase 3 | 2.43E-04 | 5.83E-07 | 1.12E-02 |
| *GFI1B* | Growth Factor Independent 1B Transcriptional Repressor | 2.63E-04 | 6.42E-07 | 1.23E-04 |
| *PNPLA6* | Patatin Like Phospholipase Domain Containing 6 | 2.65E-04 | 6.57E-07 | 4.81E-08 |
| *FAM54B* | Mitochondrial Fission Regulator 1 Like | 3.85E-04 | 9.72E-07 | 1.11E-03 |
| *C3AR1* | Complement C3a Receptor 1 | 4.07E-04 | 1.04E-06 | 4.81E-04 |
| *DSC2* | Desmocollin 2 | 4.47E-04 | 1.16E-06 | 3.02E-07 |
| *CD9* | CD9 Molecule | 4.48E-04 | 1.19E-06 | 4.27E-10 |
| *IL17RB* | Interleukin 17 Receptor B | 5.86E-04 | 1.60E-06 | 9.20E-05 |
| *KCTD15* | Potassium Channel Tetramerization Domain Containing 15 | 6.44E-04 | 1.80E-06 | 4.67E-01 |
| *SUOX* | Sulfite Oxidase | 6.59E-04 | 1.87E-06 | 5.34E-02 |
| *DAPK2* | Death Associated Protein Kinase 2 | 8.81E-04 | 2.54E-06 | 5.46E-03 |
| *STXBP5* | Syntaxin Binding Protein 5 | 1.11E-03 | 3.35E-06 | 1.64E-02 |
| *ASB2* | Ankyrin Repeat And SOCS Box Containing 2 | 1.98E-03 | 6.11E-06 | 2.18E-03 |
| *KRT81* | Keratin 81 | 2.01E-03 | 6.29E-06 | 9.90E-01 |
| *FHL3* | Four And A Half LIM Domains 3 | 2.03E-03 | 6.42E-06 | 6.95E-02 |
| *BRI3BP* | BRI3 Binding Protein | 2.14E-03 | 6.87E-06 | 1.94E-04 |
| *GAPT* | GRB2 Binding Adaptor Protein, Transmembrane | 2.15E-03 | 6.99E-06 | 5.16E-08 |
| *PAPSS1* | 3'-Phosphoadenosine 5'-Phosphosulfate Synthase 1 | 2.77E-03 | 9.11E-06 | 3.41E-05 |
| *ANKRD55* | Ankyrin Repeat Domain 55 | 2.81E-03 | 9.35E-06 | 8.79E-02 |
| *ARHGAP10* | Rho GTPase Activating Protein 10 | 2.92E-03 | 9.93E-06 | 7.98E-13 |
| *E2F5* | E2F Transcription Factor 5 | 2.92E-03 | 9.83E-06 | 7.49E-02 |
| *CD200R1* | CD200 Receptor 1 | 3.58E-03 | 1.23E-05 | 4.84E-13 |
| *SLC24A3* | Solute Carrier Family 24 Member 3 | 3.86E-03 | 1.34E-05 | 4.74E-05 |
| *PLIN2* | Perilipin 2 | 4.02E-03 | 1.42E-05 | 8.11E-06 |
| *PLEKHA7* | Pleckstrin Homology Domain Containing A7 | 5.18E-03 | 1.85E-05 | 2.39E-01 |
| *GADD45G* | Growth Arrest And DNA Damage Inducible Gamma | 5.27E-03 | 1.90E-05 | 4.65E-01 |
| *ATP6V0A2* | ATPase H+ Transporting V0 Subunit A2 | 6.63E-03 | 2.42E-05 | 4.65E-01 |
| *TRIB1* | Tribbles Pseudokinase 1 | 6.86E-03 | 2.53E-05 | 1.35E-04 |
| *GPR137B* | G Protein-Coupled Receptor 137B | 8.32E-03 | 3.10E-05 | 2.15E-04 |
| *ABCB4* | ATP Binding Cassette Subfamily B Member 4 | 8.68E-03 | 3.27E-05 | 2.41E-02 |
| *ALDH6A1* | Aldehyde Dehydrogenase 6 Family Member A1 | 9.01E-03 | 3.43E-05 | 4.44E-02 |
| *ZSCAN29* | Zinc Finger And SCAN Domain Containing 29 | 9.10E-03 | 3.50E-05 | 1.57E-01 |
| *ANXA1* | Annexin A1 | 9.82E-03 | 3.81E-05 | 7.19E-12 |
| *ASRGL1* | Asparaginase And Isoaspartyl Peptidase 1 | 1.18E-02 | 4.73E-05 | 9.91E-04 |
| *RAB40C* | RAB40C, Member RAS Oncogene Family | 1.18E-02 | 4.78E-05 | 6.48E-01 |
| *KBTBD11* | Kelch Repeat And BTB Domain Containing 11 | 1.18E-02 | 4.84E-05 | 8.20E-01 |
| *VLDLR* | Very Low Density Lipoprotein Receptor | 1.36E-02 | 5.59E-05 | 6.71E-06 |
| *PAQR7* | Progestin And AdipoQ Receptor Family Member 7 | 1.47E-02 | 6.18E-05 | 4.45E-01 |
| *SUSD1* | Sushi Domain Containing 1 | 1.47E-02 | 6.13E-05 | 1.45E-04 |
| *BIK* | BCL2 Interacting Killer | 1.59E-02 | 6.75E-05 | 6.83E-01 |
| *CXXC5* | CXXC Finger Protein 5 | 1.69E-02 | 7.30E-05 | 8.59E-01 |
| *MEIS2* | Meis Homeobox 2 | 1.69E-02 | 7.43E-05 | 3.51E-01 |
| *CACNG6* | Calcium Voltage-Gated Channel Auxiliary Subunit Gamma 6 | 1.74E-02 | 7.74E-05 | 2.43E-04 |
| *FCRLA* | Fc Receptor Like A | 1.79E-02 | 8.03E-05 | 1.71E-04 |
| *FLJ42418* | Long Intergenic Non-Protein Coding RNA 487 | 1.82E-02 | 8.32E-05 | 5.03E-01 |
| *TNNT1* | Troponin T1, Slow Skeletal Type | 1.82E-02 | 8.25E-05 | 4.11E-03 |
| *C6orf97* | Coiled-Coil Domain Containing 170 | 1.94E-02 | 8.94E-05 | 2.53E-10 |
| *CHST13* | Carbohydrate Sulfotransferase 13 | 2.25E-02 | 1.05E-04 | 9.07E-01 |
| *PKP2* | Plakophilin 2 | 2.40E-02 | 1.13E-04 | 2.75E-02 |
| *ARHGEF6* | Rac/Cdc42 Guanine Nucleotide Exchange Factor 6 | 2.41E-02 | 1.15E-04 | 1.45E-07 |
| *CD24* | CD24 Molecule | 2.50E-02 | 1.20E-04 | 1.65E-08 |
| *OLFM2* | Olfactomedin 2 | 2.72E-02 | 1.32E-04 | 1.75E-03 |
| *LRRC46* | Leucine Rich Repeat Containing 46 | 2.75E-02 | 1.34E-04 | 7.62E-01 |
| *MS4A1* | Membrane Spanning 4-Domains A1 | 2.92E-02 | 1.45E-04 | 4.93E-01 |
| *SLC4A8* | Solute Carrier Family 4 Member 8 | 3.02E-02 | 1.53E-04 | 4.91E-08 |
| *CD63* | CD63 Molecule | 3.23E-02 | 1.66E-04 | 1.36E-04 |
| *KMO* | Kynurenine 3-Monooxygenase | 3.28E-02 | 1.71E-04 | 2.19E-02 |
| *TRPC6* | Transient Receptor Potential Cation Channel Subfamily C Member 6 | 3.28E-02 | 1.70E-04 | 2.04E-03 |
| *P2RY14* | Purinergic Receptor P2Y14 | 3.93E-02 | 2.06E-04 | 5.18E-10 |
| *TCL1A* | T Cell Leukemia/Lymphoma 1A | 4.18E-02 | 2.21E-04 | 1.74E-01 |
| *ADAM8* | ADAM Metallopeptidase Domain 8 | 4.30E-02 | 2.29E-04 | 5.53E-02 |

**Supplementary Table 5. Genes mapped to drug compounds.** The associations of genes and drug compounds are from DGIdb.

| Gene | Drug |
| --- | --- |
| *ADORA3* | ADENOSINE |
| *ANXA1* | DEXAMETHASONE |
| *IL5RA* | BENRALIZUMAB |
| *ANXA1* | HYDROCORTISONE |
| *ADORA3* | NAMODENOSON |
| *ADORA3* | IB-MECA |
| *ANXA1* | AMCINONIDE |
| *PTGDR2* | AM-211 |
| *PTGDR2* | OC000459 |
| *ADORA3* | CAFFEINE |
| *ADORA3* | AMINOPHYLLINE |
| *ADORA3* | N6-CYCLOPENTYLADENOSINE |
| *ADORA3* | NECA |
| *ADORA3* | CHEMBL382194 |
| *ADORA3* | CHROMOCARB |
| *ANXA1* | METHYLPREDNISOLONE |
| *ANXA1* | STAUROSPORINE |
| *C3AR1* | CHEMBL389348 |
| *CAT* | ALCOHOL |
| *FBP1* | MB-07803 |
| *PTGDR2* | AZD1981 |
| *PTGDR2* | SETIPIPRANT |
| *PTGDR2* | MK-7246 |
| *PTGDR2* | VIDUPIPRANT |
| *PTGDR2* | QAV680 |
| *PTGDR2* | AM-461 |
| *PTGDR2* | RAMATROBAN |
| *PTGDR2* | ADC3680 |
| *PTGDR2* | ARRY-502 |
| *IDO1* | EPACADOSTAT |
| *IDO1* | PHENYLHYDRAZINE |
| *IDO1* | CHEMBL1933308 |
| *IDO1* | CHEMBL1224312 |
| *IDO1* | CHEMBL14145 |
| *IDO1* | 2-MERCAPTOBENZOTHIAZOLE |
| *IDO1* | CHEMBL1668301 |
| *INPP1* | LITHIUM |
| *SLC4A8* | ALCOHOL |
| *THBS4* | VASOPRESSIN |
| ACACB | PF-05175157 |
| ACACB | METFORMIN |
| ADORA3 | CAFFEINE |
| ADORA3 | THEOPHYLLINE |
| *ADORA3* | THEOPHYLLINE SODIUM GLYCINATE |
| *ADORA3* | PICLIDENOSON |
| *ADORA3* | TONAPOFYLLINE |
| *ADORA3* | OXTRIPHYLLINE |
| *ADORA3* | QAF805 |
| *ADORA3* | CHEMBL136689 |
| *ADORA3* | VANOXERINE |
| *ADORA3* | CIPARGAMIN |
| *ANXA1* | CLOBETASOL |
| *ANXA1* | PREDNISOLONE |
| *ANXA1* | DESOXIMETASONE |
| *ANXA1* | PREDNISONE |
| *ANXA1* | BETAMETHASONE |
| *ANXA1* | PREDNICARBATE |
| *ANXA1* | ALCLOMETASONE |
| *ANXA1* | BECLOMETHASONE |
| *ANXA1* | TRIAMCINOLONE |
| *ANXA1* | MOMETASONE |
| *ANXA1* | CLOCORTOLONE |
| *ANXA1* | RIMEXOLONE |
| *ANXA1* | DIFLORASONE |
| *ANXA1* | HYDROCORTAMATE |
| *ANXA1* | DESONIDE |
| *BACE2* | PHENSERINE |
| *CACNG6* | GABAPENTIN |
| *CACNG6* | PREGABALIN |
| *CACNG6* | IMAGABALIN |
| *CACNG6* | BEPRIDIL HYDROCHLORIDE |
| *CACNG6* | GABAPENTIN ENACARBIL |
| *CACNG6* | ATAGABALIN |
| *CAMK1* | LINIFANIB |
| *CAMK1* | LAUROGUADINE |
| *CAMK1* | ILORASERTIB |
| *CLC* | CHLOROTOXIN |
| *CLC* | MOXIDECTIN |
| *CLC* | TOZULERISTIDE |
| *EEF2K* | LAPATINIB |
| *PTGDR2* | MK-1029 |
| *PTGDR2* | FEVIPIPRANT |
| *PTGDR2* | LAROPIPRANT |
| *PTGDR2* | RG7185 |
| *PTGDR2* | DONITRIPTAN |
| *IDO1* | LINRODOSTAT |
| IDO1 | PF-06840003 |
| IDO1 | TRYPTOPHAN |

**Supplementary Table 6.** Association results in the FHS for the SNPs with the strongest evidence of association with IgE levels (n=42), with a p<5×10^-8^ (threshold for genome-wide significance).

| Nearest Gene | SNP | Chr | Position | Minor Allele | iMAF | FHS Beta | FHS P-value |
| --- | --- | --- | --- | --- | --- | --- | --- |
|  | rs2251746 | 1 | 159272060 | T | 0.73 | 0.086 | 2.12E-13 |
| *FCER1A* | rs2427837 | 1 | 159258545 | G | 0.73 | 0.080 | 5.54E-12 |
|  | rs2427823 | 1 | 159264017 | G | 0.70 | 0.091 | 1.42E-11 |
| *OR10J3* | rs6703348 | 1 | 159291683 | C | 0.75 | 0.081 | 2.11E-11 |
| *OR10J3* |  | 1 | 159287343 | R | 0.75 | 0.081 | 2.14E-11 |
| *FCER1A* | rs2262422 | 1 | 159223787 | G | 0.76 | 0.084 | 3.33E-11 |
| *FCER1A* | rs2427831 | 1 | 159222847 | T | 0.73 | 0.077 | 9.38E-11 |
| *FCER1A* | rs2511211 | 1 | 159230471 | T | 0.77 | 0.081 | 3.10E-10 |
| *DARC* | rs863016 | 1 | 159203933 | A | 0.73 | 0.074 | 5.10E-10 |
|  | rs1059513 | 12 | 57489709 | T | 0.89 | 0.147 | 1.09E-09 |
|  | rs3024971 | 12 | 57493727 | T | 0.89 | 0.149 | 1.28E-09 |
|  | rs2494264 | 1 | 159266838 | T | 0.54 | 0.063 | 1.49E-09 |
|  | rs2247584 | 1 | 159259680 | T | 0.54 | 0.062 | 2.53E-09 |
| *FCER1A* | rs2427836 | 1 | 159254764 | T | 0.54 | 0.062 | 2.65E-09 |
| *FCER1A* | rs2511208 | 1 | 159223834 | G | 0.54 | 0.063 | 3.03E-09 |
| *FCER1A* | rs2325928 | 1 | 159239577 | C | 0.54 | 0.062 | 3.38E-09 |
| *FCER1A* | rs2494256 | 1 | 159229537 | G | 0.54 | 0.062 | 3.56E-09 |
|  | rs1295686 | 5 | 131995843 | C | 0.80 | -0.079 | 3.64E-09 |
| *OR10J3* |  | 1 | 159282991 | R | 0.56 | 0.063 | 3.68E-09 |
| *FCER1A* | rs2325919 | 1 | 159222811 | G | 0.54 | 0.062 | 4.33E-09 |
| *OR10J3* | rs12724895 | 1 | 159291045 | T | 0.56 | 0.062 | 4.60E-09 |
| *OR10J3* | rs12028986 | 1 | 159300662 | G | 0.56 | 0.062 | 4.65E-09 |
| *OR10J3* | rs11265171 | 1 | 159302180 | G | 0.56 | 0.061 | 5.99E-09 |
| *OR10J3* | rs2494251 | 1 | 159281151 | G | 0.55 | 0.061 | 6.65E-09 |
|  | rs848 | 5 | 131996500 | C | 0.80 | -0.078 | 7.23E-09 |
| *OR10J3* | rs11265166 | 1 | 159288468 | G | 0.55 | 0.061 | 7.69E-09 |
| *OR10J3* | rs4656784 | 1 | 159326880 | A | 0.78 | 0.077 | 7.94E-09 |
| *FCER1A* | rs2494257 | 1 | 159230098 | T | 0.54 | 0.061 | 1.05E-08 |
| *BATF3* | rs17019567 | 1 | 212855923 | A | 0.92 | 0.111 | 1.27E-08 |
| *OR10J1* | rs12118201 | 1 | 159396941 | C | 0.79 | 0.075 | 1.35E-08 |
| *BATF3* | rs61829183 | 1 | 212847352 | C | 0.92 | 0.112 | 1.39E-08 |
|  | rs1295685 | 5 | 131996445 | G | 0.82 | -0.077 | 1.44E-08 |
|  | rs20541 | 5 | 131995964 | G | 0.81 | -0.076 | 1.49E-08 |
|  | rs847 | 5 | 131996669 | C | 0.81 | -0.077 | 1.63E-08 |
| *OR10J1* | rs34448772 | 1 | 159372815 | A | 0.78 | 0.073 | 1.63E-08 |
|  | rs116388438 | 6 | 29913344 | G | 0.79 | -0.084 | 2.21E-08 |
|  |  | 12 | 57464685 | R | 0.90 | 0.126 | 2.22E-08 |
|  | rs113413483 | 6 | 29911203 | A | 0.75 | -0.107 | 3.24E-08 |
| *TMEM194A* | rs11172086 | 12 | 57449206 | C | 0.90 | 0.121 | 3.68E-08 |
|  | rs115270926 | 6 | 29912348 | A | 0.79 | -0.091 | 3.94E-08 |
| *OR10J1* | rs61821493 | 1 | 159411492 | T | 0.75 | 0.065 | 4.32E-08 |
| *OR10J1* | rs7540542 | 1 | 159430327 | G | 0.75 | 0.066 | 4.85E-08 |

**Supplementary Table 7.** MR results for genes putatively causal for allergic diseases at Bonferroni-corrected p<2.70×10^-4^ (n=71).

| Gene Symbol | Transcript # | Method | nsnp | Beta | SE | P-Value |
| --- | --- | --- | --- | --- | --- | --- |
| *GPI* | 3829687 | Wald ratio | 1 | 23.34 | 0.52 | 0.00E+00 |
| *PGD* | 2319802 | Wald ratio | 1 | 69.40 | 0.71 | 0.00E+00 |
| *P4HA1* | 3294159 | Wald ratio | 1 | 11.13 | 0.24 | 0.00E+00 |
| *NDFIP2* | 3495076 | Wald ratio | 1 | 77.71 | 0.79 | 0.00E+00 |
| *GPR114* | 3662774 | Wald ratio | 1 | -61.44 | 0.72 | 0.00E+00 |
| *CPT1A* | 3379644 | Wald ratio | 1 | 35.94 | 0.47 | 0.00E+00 |
| *ZNF610* | 3840164 | Wald ratio | 1 | -50.84 | 0.57 | 0.00E+00 |
| *ABTB2* | 3368940 | Wald ratio | 1 | 49.07 | 0.52 | 0.00E+00 |
| *CD9* | 3402315 | Wald ratio | 1 | 25.70 | 0.44 | 0.00E+00 |
| *STK17A* | 2999485 | Wald ratio | 1 | 42.33 | 0.55 | 0.00E+00 |
| *SMPD3* | 3696317 | Wald ratio | 1 | -59.72 | 0.80 | 0.00E+00 |
| *GCNT1* | 3175494 | Wald ratio | 1 | 58.88 | 0.62 | 0.00E+00 |
| *CCR4* | 2616131 | Wald ratio | 1 | 19.86 | 0.25 | 0.00E+00 |
| *KLHL6* | 2708066 | Wald ratio | 1 | -22.56 | 0.52 | 0.00E+00 |
| *PRF1* | 3293435 | Wald ratio | 1 | 38.14 | 0.75 | 0.00E+00 |
| *ATP8B2* | 2360206 | Wald ratio | 1 | 47.81 | 0.56 | 0.00E+00 |
| *GRB10* | 3050462 | Wald ratio | 1 | 29.50 | 0.72 | 0.00E+00 |
| *ZMYND11* | 3231389 | Wald ratio | 1 | -73.02 | 0.78 | 0.00E+00 |
| *OLIG1* | 3918429 | Wald ratio | 1 | 96.66 | 1.05 | 0.00E+00 |
| *FBP1* | 3215570 | Wald ratio | 1 | -60.73 | 0.93 | 0.00E+00 |
| *ORM2* | 3186137 | Wald ratio | 1 | -30.27 | 0.31 | 0.00E+00 |
| *SAMSN1* | 3925473 | Wald ratio | 1 | 51.60 | 0.65 | 0.00E+00 |
| *FCRL2* | 2439052 | Wald ratio | 1 | -53.35 | 0.65 | 0.00E+00 |
| *CCDC86* | 3332548 | Wald ratio | 1 | 57.82 | 0.63 | 0.00E+00 |
| *KIT* | 2727587 | Wald ratio | 1 | 88.05 | 0.91 | 0.00E+00 |
| *TUBGCP4* | 3591400 | Wald ratio | 1 | -15.39 | 0.20 | 0.00E+00 |
| *PLD3* | 3833443 | Wald ratio | 1 | 40.43 | 0.69 | 0.00E+00 |
| *BHLHE40* | 2608725 | Wald ratio | 1 | -24.26 | 0.39 | 0.00E+00 |
| *THUMPD1* | 3683783 | Wald ratio | 1 | 31.68 | 0.32 | 0.00E+00 |
| *CLINT1* | 2883609 | Wald ratio | 1 | 58.41 | 0.68 | 0.00E+00 |
| *IL5RA* | 2660617 | Wald ratio | 1 | 44.00 | 0.48 | 0.00E+00 |
| *IL2RA* | 3275729 | Wald ratio | 1 | 28.62 | 0.31 | 0.00E+00 |
| *SRGAP3* | 2662087 | Wald ratio | 1 | 79.91 | 0.84 | 0.00E+00 |
| *NUPL1* | 3482219 | Wald ratio | 1 | 52.73 | 1.00 | 0.00E+00 |
| *NUP93* | 3662265 | Wald ratio | 1 | 52.85 | 0.38 | 0.00E+00 |
| *IL17RB* | 2624565 | Wald ratio | 1 | -33.26 | 0.35 | 0.00E+00 |
| *CRIP1* | 3554851 | Wald ratio | 1 | 28.70 | 0.33 | 0.00E+00 |
| *GPR137B* | 2386747 | Wald ratio | 1 | -63.66 | 0.65 | 0.00E+00 |
| *ID2* | 2468622 | Wald ratio | 1 | -38.40 | 0.41 | 0.00E+00 |
| *CLCNKB* | 2322264 | Wald ratio | 1 | 68.46 | 0.73 | 0.00E+00 |
| *PLAC4* | 3932917 | Wald ratio | 1 | -15.36 | 0.17 | 0.00E+00 |
| *SYNE1* | 2979871 | Wald ratio | 1 | -44.19 | 0.84 | 0.00E+00 |
| *FCRLA* | 2363852 | Wald ratio | 1 | -66.26 | 0.67 | 0.00E+00 |
| *COBLL1* | 2584787 | Wald ratio | 1 | 84.02 | 1.07 | 0.00E+00 |
| *INPP5A* | 3272205 | Wald ratio | 1 | -22.36 | 0.39 | 0.00E+00 |
| *SEMA7A* | 3632907 | Wald ratio | 1 | -47.78 | 0.91 | 0.00E+00 |
| *PPM1L* | 2650393 | Wald ratio | 1 | -11.57 | 0.27 | 0.00E+00 |
| *GATA3* | 3234277 | Wald ratio | 1 | 42.24 | 0.50 | 0.00E+00 |
| *ADORA3* | 2427981 | Inverse variance weighted | 2 | 20.17 | 0.57 | 3.69E-278 |
| *BRI3BP* | 3436544 | Wald ratio | 1 | 31.68 | 0.89 | 1.23E-277 |
| *SEMA5A* | 2847967 | Wald ratio | 1 | 37.37 | 1.12 | 1.49E-244 |
| *HRASLS2* | 3376512 | Wald ratio | 1 | 10.94 | 0.33 | 1.48E-236 |
| *PMP22* | 3746574 | Inverse variance weighted | 2 | 30.20 | 1.15 | 1.04E-150 |
| *PDE8A* | 3606034 | Wald ratio | 1 | 23.30 | 0.93 | 9.36E-138 |
| *VKORC1L1* | 3005280 | Wald ratio | 1 | 23.62 | 1.12 | 4.80E-99 |
| *CD63* | 3457160 | Inverse variance weighted | 2 | -79.45 | 6.16 | 4.45E-38 |
| *TNIK* | 2705266 | Inverse variance weighted | 3 | -70.60 | 9.60 | 1.93E-13 |
| *SLC35D1* | 2417095 | Inverse variance weighted | 3 | 14.70 | 2.16 | 9.56E-12 |
| *FMNL3* | 3454006 | Inverse variance weighted | 2 | -38.64 | 6.15 | 3.33E-10 |
| *P2RY14* | 2701033 | Inverse variance weighted | 4 | 15.81 | 2.75 | 8.76E-09 |
| *VSTM1* | 3870449 | Inverse variance weighted | 3 | -6.93 | 1.21 | 1.08E-08 |
| *TNFRSF9* | 2395146 | Inverse variance weighted | 2 | -11.92 | 2.21 | 6.87E-08 |
| *VLDLR* | 3160175 | Inverse variance weighted | 5 | 33.30 | 7.04 | 2.24E-06 |
| *CCL23* | 3753985 | Inverse variance weighted | 2 | -36.45 | 7.78 | 2.83E-06 |
| *KLF6* | 3274361 | Inverse variance weighted | 2 | -54.79 | 12.96 | 2.36E-05 |
| *ABCC1* | 3649890 | Inverse variance weighted | 2 | -45.65 | 10.81 | 2.41E-05 |
| *CASP3* | 2796484 | Inverse variance weighted | 2 | -11.06 | 2.76 | 6.12E-05 |
| *TEC* | 2768396 | Inverse variance weighted | 3 | -22.77 | 5.84 | 9.64E-05 |
| *INPP1* | 2520113 | Inverse variance weighted | 2 | -25.51 | 6.58 | 1.07E-04 |
| *GZMB* | 3558375 | Inverse variance weighted | 2 | -13.78 | 3.57 | 1.12E-04 |
| *SLC7A8* | 3557209 | Inverse variance weighted | 3 | -35.83 | 9.74 | 2.34E-04 |

**Supplementary Table 8. Expression levels of the four genes (*CLC, S100A13, CDC21* and *GCNT1*) in lung tissues from single-cell RNA-seq.** [The single-cell data were from the Human Protein Atlas](file:///Users/krecto/Downloads/The%20single-cell%20data%20were%20from%20the%20Human%20Protein%20Atlas) (<https://www.proteinatlas.org/>). The gene expression value is the normalized transcript per million (nTPM).

|  | *CLC* | *S100A13* | *CCDC21* | *GCNT1* |
| --- | --- | --- | --- | --- |
| Macrophages c-0 | 0 | 227.5 | 5.6 | 32.1 |
| Alveolar cells type 2 c-1 | 0 | 118.2 | 2.1 | 1.4 |
| Macrophages c-2 | 0 | 13.4 | 4.9 | 8.5 |
| T-cells c-3 | 0 | 9.1 | 4.5 | 1.5 |
| Granulocytes c-4 | 0 | 20.2 | 5 | 12.6 |
| Fibroblasts c-5 | 0 | 91.6 | 2.2 | 4.5 |
| Alveolar cells type 2 c-6 | 0 | 73.1 | 2.4 | 2.4 |
| Club cells c-7 | 0 | 101.5 | 4.9 | 9.9 |
| Respiratory ciliated cells c-8 | 0 | 65.6 | 1.5 | 10.2 |
| Endothelial cells c-9 | 0 | 99.8 | 3.1 | 0 |
| Alveolar cells type 1 c-10 | 0 | 28.9 | 0 | 0 |
